# Disruption of STAT5b-Regulated Sexual Dimorphism of the Liver Transcriptome by Diverse Factors Is a Common Event

**DOI:** 10.1371/journal.pone.0148308

**Published:** 2016-03-09

**Authors:** Keiyu Oshida, Naresh Vasani, David J. Waxman, J. Christopher Corton

**Affiliations:** 1 Integrated Systems Toxicology Division, NHEERL/ORD, US-EPA, Research Triangle Park, NC 27711, United States of America; 2 Division of Cell and Molecular Biology, Department of Biology and Bioinformatics Program, Boston University, Boston, MA 02215, United States of America; University of Nebraska Medical Center, UNITED STATES

## Abstract

Signal transducer and activator of transcription 5b (STAT5b) is a growth hormone (GH)-activated transcription factor and a master regulator of sexually dimorphic gene expression in the liver. Disruption of the GH hypothalamo-pituitary-liver axis controlling STAT5b activation can lead to metabolic dysregulation, steatosis, and liver cancer. Computational approaches were developed to identify factors that disrupt STAT5b function in a mouse liver gene expression compendium. A biomarker comprised of 144 STAT5b-dependent genes was derived using comparisons between wild-type male and wild-type female mice and between STAT5b-null and wild-type mice. Correlations between the STAT5b biomarker gene set and a test set comprised of expression datasets (biosets) with known effects on STAT5b function were evaluated using a rank-based test (the Running Fisher algorithm). Using a similarity p-value ≤ 10^−4^, the test achieved a balanced accuracy of 99% and 97% for detection of STAT5b activation or STAT5b suppression, respectively. The STAT5b biomarker gene set was then used to identify factors that activate (masculinize) or suppress (feminize) STAT5b function in an annotated mouse liver and primary hepatocyte gene expression compendium of ~1,850 datasets. Disruption of GH-regulated STAT5b is a common phenomenon in liver in vivo, with 5% and 29% of the male datasets, and 11% and 13% of the female datasets, associated with masculinization or feminization, respectively. As expected, liver STAT5b activation/masculinization occurred at puberty and suppression/feminization occurred during aging and in mutant mice with defects in GH signaling. A total of 70 genes were identified that have effects on STAT5b activation in genetic models in which the gene was inactivated or overexpressed. Other factors that affected liver STAT5b function were shown to include fasting, caloric restriction and infections. Together, these findings identify diverse factors that perturb the hypothalamo-pituitary-liver GH axis and disrupt GH-dependent STAT5b activation in mouse liver.

## Introduction

Signal transducer and activator of transcription 5b (STAT5b) is one of seven mammalian STAT transcription factors [1, 2]. Like other family members, STAT5b responds to a variety of extracellular cytokine and growth factor signals, including interleukins, epidermal growth factor and growth hormone (GH) [3–5]. GH is secreted by the pituitary gland in a sex-dependent manner under the control of gonadal steroids [6, 7]. These steroids regulate pituitary GH secretion at the level of neurosecretory cells of the hypothalamus, which release into venous blood surrounding the pituitary a GH-releasing hormone (GHRH or somatocrinin) and a GH release-inhibitory hormone (GHIH or somatostatin). The balance of these two peptide hormones, which determines the temporal patterns of pituitary GH secretion, is affected by physiological stimulators (e.g., exercise, nutrition, sleep) and inhibitors (e.g., free fatty acids, glucose) [8], in addition to gonadal steroids [9]. GH secretion is under feedback inhibitory control by insulin-like growth factor 1 (IGF-1), which is induced in the liver following GH activation of STAT5b [10]. The sex-dependent patterns of pituitary GH secretion impart substantial sex differences to GH-regulated functions, especially gene expression in the liver [11].

Sexual dimorphism of the liver has significant metabolic consequences. Xenobiotic clearance of a wide variety of drugs and toxins differs between women and men due, in part, to sex-biased expression of enzymes, such as cytochrome P450 3A4 [12–15]. Rats and mice also exhibit pronounced liver sex-biased expression of xenobiotic metabolism enzymes [16]. The hypothalamic-pituitary-liver (HPL) axis is an important determinant of sex-dependent liver functions. In rodentia and to a lesser extent in humans, the pattern of GH secretion from the anterior pituitary differs between the sexes. In rats and mice, plasma GH levels are highly pulsatile in males, with hormone peaks followed by a well-defined GH-free interval. In females, pituitary GH release occurs more frequently, and GH is present in the plasma in a more continuous manner [6, 8]. These sex-dependent plasma GH profiles, in turn, regulate liver gene expression at the level of transcription, as demonstrated for several GH-regulated liver cytochrome P450 (*Cyp*) genes (for review, see Waxman and O’Connor, 2006 [17]).

STAT5b is activated by tyrosine phosphorylation catalyzed by Janus Kinase 2 (JAK2), a GH receptor-associated tyrosine kinase. GH binding to its cell surface receptor (GHR) activates JAK2, leading to phosphorylation of the GH receptor by JAK2 on multiple cytoplasmic domain tyrosine residues, several of which serve as docking sites for STAT5b [4]. JAK2 then phosphorylates STAT5b on Tyr 699, which enables STAT5b to dimerize, translocate to the nucleus, and bind to thousands of STAT5 response elements in regulated genes [18] through its unique immunoglobulin-fold DNA-binding domain [19]. STAT5b is directly activated in male rat and mouse liver in response to each incoming plasma GH pulse, whereas in females, the persistence of plasma GH stimulation leads to persistence of liver STAT5b signaling [20], which in the female rat is at a substantially lower level than seen in males [21–24]. The hypothesis that STAT5b is the major mediator of the sex-dependent effects of GH on liver gene expression is supported by work in STAT5b-deficient male mice, which display a reduced body growth rate at puberty and a loss of sex-specific liver gene expression affecting many genes that control the metabolism and transport of exogenous chemicals [16, 25, 26].

STAT5b function can be modulated by chemical exposures, including those that are environmentally relevant, as indicated by the inhibitory cross-talk between STAT5b and the nuclear receptors PPARα and PPARγ when activated by foreign chemicals [27, 28]. However, little is known about the universe of chemicals that may disrupt STAT5b function, prompting us to develop the broad approach described here to identify factors that disrupt STAT5b function and its control by the HPL-GH axis. Given the role of STAT5b as a mediator of sex-specific transcriptional effects on phase I and II metabolism and transporter gene expression [11], foreign chemicals and other factors that modulate STAT5b function could impact the ability of the liver to metabolize and eliminate xenobiotics, with effects on toxicity in liver and other organs. Further, disruption of pituitary GH secretion and the HPL axis results in adverse effects that overlap with those induced by xenobiotics, including fatty liver disease, obesity and cancer [29].

Methods that accurately predict changes in hepatic STAT5b function, including activation and suppression of STAT5b activity, would help to identify factors that disrupt the HPL-GH axis. Given the requirement for an intact HPL axis for the physiologically relevant actions of STAT5b in the liver, in vitro assays that recapitulate the male-specific pattern of STAT5b activation have been difficult to develop. In the present study, computational methods were developed to evaluate effects of diverse stressors on STAT5b activation in the livers of mice, as assessed using microarray-derived gene lists. A STAT5b-specific biomarker comprised of genes that exhibit sexual dimorphism was constructed based on wild-type male vs. wild-type female and STAT5b-null vs. STAT5b-wild-type mouse liver microarray comparisons. Using a fold-change rank-based statistical test, the STAT5b biomarker gene set was found to be highly accurate in predicting activation or suppression of STAT5b function. This computational approach was used to screen a gene expression compendium of ~1,850 comparisons to identify diets, infections and genes that activate or suppress liver STAT5b activity and that are proposed to disrupt STAT5b function in mouse liver in vivo. The effects of exposure to hormones and chemicals on STAT5b are described in an accompanying publication [30].

## Methods

### Strategy for identification of factors that alter liver STAT5b function

**Fig 1:**
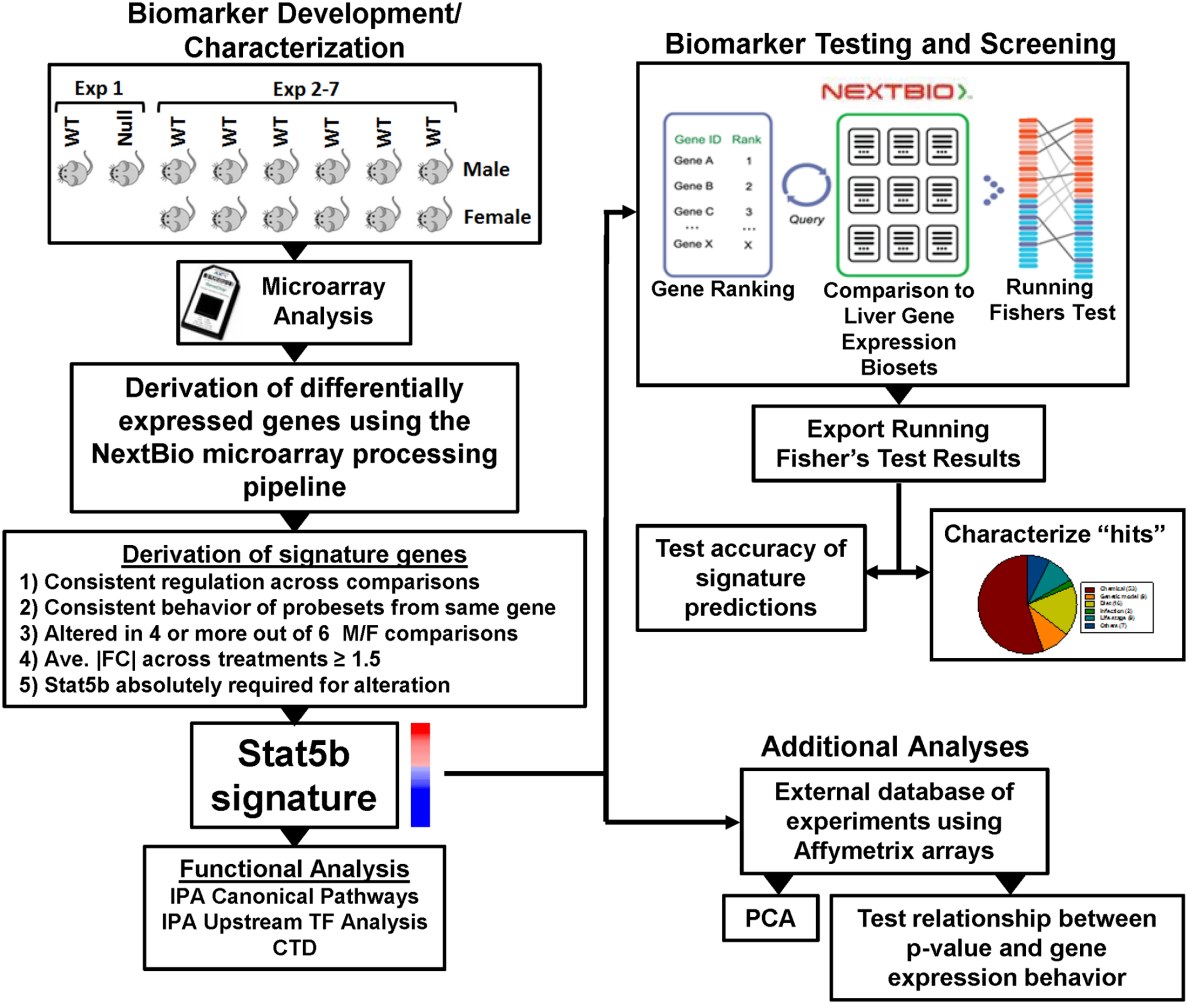
Scheme for STAT5b biomarker development and screening of a mouse liver gene expression compendium. *Left*, STAT5b biomarker development and characterization. Experiments used to identify STAT5b-regulated genes included a comparison of male STAT5b-null mice and male wild-type mice [16] (experiment 1) and comparisons between male and female mice from experiments 2–7 (GSE21048 [39], GSE7169 [40], GSE5072 [16], GSE54597 [41], GSE7170[40], and GSE21065 [42]). Differentially expressed genes (DEGs) were identified using the NextBio microarray processing pipeline. Biomarker genes were identified from the DEGs after applying the 5 filtering steps listed. Genes in the biomarker were evaluated by Ingenuity Pathway Analysis (IPA) for canonical pathway enrichment and potential transcription factor regulators and by the Comparative Toxicogenomics Database (CTD) to evaluate literature evidence for consistent regulation of biomarker genes by hormones. *Right*, biomarker testing and screening. The STAT5b biomarker gene set was imported into the NextBio environment, in which internal protocols rank-ordered the genes based on their fold-change. Screening was carried out by comparison of the biomarker gene set to each bioset in the NextBio database using a pair-wise rank-based enrichment algorithm (the Running Fisher algorithm). The results of the comparisons, including the direction of correlation and p-value for each bioset in the compendium, were exported and used to populate a master table containing bioset experimental details. A test of the accuracy of the biomarker gene set predictions was carried out using treatments that are known positives and negatives for STAT5b activation. Screening “hits” were characterized in terms of the factors that impact STAT5b function. Additionally, an external gene expression database of experiments performed with Affymetrix gene chips was used for principal components analysis (PCA) and to assess the relationship between p-value and behavior of the genes in the biomarker. One part of this figure was adapted from a figure in Kupershmidt et al. [34]. The figure is similar to that in Oshida[31–33].

The methods used in this study are outlined in Fig 1. A gene expression biomarker comprised of STAT5b-dependent genes was developed using an annotated database of gene expression profiles of statistically filtered genes (also called biosets). The STAT5b biomarker gene set is a list of sex differentially expressed hepatic genes that require STAT5b for their sex differences, together with their associated fold-change values, which correspond to average differences across studies in expression between male and female mouse liver. A commercially available gene expression database provided by NextBio (www.nextbio.com) was used. The NextBio database contains over 117,000 lists of statistically filtered genes from over 17,500 microarray studies carried out in 16 species (as of January, 2015). Each list (bioset) was compared to all other biosets in the database using a fold-change rank-based statistical algorithm called the Running Fisher test, which allows an assessment of the overlap in regulated genes and whether those overlapping genes are regulated in a similar or opposite manner. In this study, only biosets from mouse liver, mouse primary hepatocytes and hepatocyte-derived cell lines were evaluated. Available information about each bioset was extracted from the NextBio database and used to populate a compendium of information about each of the experiments. A subset of biosets was further annotated using information derived from the original publication or its Gene Expression Omnibus (GEO) submission. To facilitate comparisons across the compendium, each bioset was annotated for the type of factor (e.g., diet) and the name of the factor (e.g., fasting) examined. To assess activation or suppression of STAT5b function, the STAT5b biomarker gene set was uploaded to the NextBio database and compared to all biosets in the database using the Running Fisher algorithm. Results of the comparisons were exported and used to populate the annotated compendium with p-values for each comparison. Test results were used to determine the accuracy of predictions as described below and to characterize those biosets that achieved statistical significance. Additional analyses were carried out using an independent database of experiments using Affymetrix mouse arrays. We have previously used this analysis strategy to accurately identify factors that activate or suppress other transcription factors (AhR, CAR and PPARα) [31–33].

### Identification of differentially expressed genes in NextBio microarray datasets

Strict criteria for inclusion of experiments in the database are applied by NextBio. In their initial description of the database, Kupershmidt et al. (2010) [34]collected and analyzed over 6,000 individual experiments comprised of more than 140,000 individual samples from different public sources of large-scale experimental data. Out of these experiments, only ~4,000 passed the quality control (QC) criteria. Reasons for exclusion included insufficient replicates, lack of control samples, unsupported platforms (e.g., platforms that do not cover more than half the number of genes for an organism), or inadequate QC metrics during pre-processing and differential expression analysis. As of September, 2014 ~30% of the studies of all studies analyzed have been excluded (NextBio, personal communication).

All differentially regulated genes were identified using the following criteria as part of the NextBio pipeline, as described in greater detail in Kuperschmidt et al. (2010) [34]. Datasets which passed the initial QC criteria went through processing steps that depended on the data type, platform, and experimental design used to generate the data. These steps included 1) background subtraction, if applicable, 2) expression summarization, e.g., using robust multichip average (RMA) when Affymetrix.cel file data was used, 3) data transformation (to log space), technical replicate averaging, and negative value correction, 4) normalization—RMA, per-chip median, or Lowess where applicable, 5) quality control assessment, and 6) statistical (differential expression) analysis. For Affymetrix-based studies in which.cel files were available, RMA normalization was applied [35]. Otherwise, expression summary intensities, such as those generated by MAS5 (Affymetrix) or dChip, were processed [36]. The vast majority of processed data in NextBio consists of case-control experimental design analyzed using Welch or standard t-tests, paired or unpaired, as appropriate. Quality assessment methods were employed to review sample-level and dataset-level integrity—these included curator review of pre- and post-normalization boxplots, missing value counts, and p-value histograms (after statistical testing) with false discovery rate analysis to determine whether the number of significantly changing genes is greater than expected by chance. To obtain the final list of differentially-expressed genes, a p-value significance cutoff of 0.05 (without any multiple testing correction) and a minimum absolute fold-change cutoff of 1.2 (typically the lowest sensitivity threshold of commercial microarray platforms) was used. This double filtering procedure addresses different aspects of variability in the data [34]. Genes with signals lower than a 20^th^ percentile cutoff in both control and test groups were discarded to address the potential unreliability of the fold-change metric at low intensity levels [37]. The thresholds set in this analysis pipeline are intentionally permissive to ensure that biosets contain all potentially interesting genes. It should be noted that the potential for introducing noise, i.e., more false positives, is balanced by applying the basic quality control metrics described above and by incorporating a normalized rank-based scheme that captures the relative importance of each gene in a bioset.

### Identification of differentially expressed genes in an external database

Independent of the NextBio database, a database of gene expression changes was assembled by our group using comparisons from mouse liver, mouse primary hepatocytes and hepatocyte-derived cell lines. All of these experiments were conducted using Affymetrix microarrays. Cel files were downloaded from publicly available sources including GEO and ArrayExpress and first analyzed by Bioconductor SimpleAffy [38] to assess sample quality. Cel files from individual studies were normalized using Rosetta Resolver^®^ version 7.1 Affymetrix Rosetta-Intensity Profile Builder software (Rosetta Inpharmatics, Kirkland, WA). Statistically significant genes were identified by one-way ANOVA with a false discovery rate (Benjamini-Hochberg test) of ≤ 0.01. A total of ~890 biosets were created and annotated as described below using information from the original study. Master tables were built using the common probe sets shared by Affymetrix 430A or 430_2 chip types. The filtered fold-change table was used to evaluate the relationships between the p-value from the Running Fisher test and the expression behavior of the genes in the biomarker (Fig 1). An unfiltered fold-change table was used in the Principal Components Analysis described below. All statistically filtered gene lists were uploaded into NextBio after filtering for │fold-change│ ≥ 1.2.

### Annotation of a mouse liver gene expression compendium

Annotation has been described in our previous studies [31–33]. “All of the biosets examining gene expression in mouse liver, mouse primary hepatocytes, or mouse hepatocyte-derived cell lines were annotated for study characteristics allowing a systematic assessment of the effect of different factors on STAT5b function. The list of descriptors provided for each of the biosets included study ID (i.e., source), name, classification of factor (e.g., chemical, diet, genotype, etc.), name of chemical or treatment, CAS# (where appropriate), sex, source of material (e.g., liver or hepatocyte), microarray type, and type of genetic model used (e.g., wild-type, nullizygous, transgenic).” [32]. Assignments of sex were made from the GEO or ArrayExpress submission or from the original paper. Given the importance of sex in determining gene expression and phenotypic responses, it was surprising that many of the published studies did not provide any information about the sex of the animals used (i.e., E-MEXP-834, E-MEXP-835, E-MEXP-856, GSE1093, GSE11098, GSE11116, GSE11685, GSE11899, GSE13992, GSE14539, GSE17730, GSE19675, GSE23088, GSE25142, GSE25457, GSE3129, GSE3150, GSE32354, GSE34285, GSE34838, GSE36307, GSE39313, GSE6903, GSE8642, GSE9484, GSE9892). Further details of each experiment not in our annotated compendium are available at GEO (http://www.ncbi.nlm.nih.gov/geo/) or ArrayExpress (http://www.ebi.ac.uk/arrayexpress/).

### Identification of STAT5b biomarker genes

Methods for biomarker development are outlined in Fig 1, **left**. STAT5b biomarker genes were identified in two steps. First, comparisons were made between statistically-filtered gene lists between control males and control females from the studies GSE21048 [39], GSE7169 [40], GSE5072 [16], GSE54597 [41], GSE7170 [40] and GSE21065 [42]. Statistically-filtered genes were derived as described above using the NextBio microarray processing pipeline. Biomarker genes were selected based on the following criteria: 1) exhibited statistically significant changes between males and females in 4 or more of the 6 comparisons; 2) exhibited the same direction of change in each of the male to female comparisons; and 3) the average │fold-change│ ≥ 1.5 across the comparisons. Second, the genes with consistent regulation across the 6 studies were compared to genes differentially regulated by STAT5b to exclude genes that were not also altered between male STAT5b-null mice and male wild-type mice [16] i.e., genes whose expression was STAT5b-independent. We thus identified a STAT5b biomarker comprised of 144 genes (74 with increased expression and 70 with decreased expression) that exhibited similar regulation in male vs. female comparisons, all of which were dependent on STAT5b for sex-specific expression. The STAT5b biomarker gene set was imported into NextBio without any further filtering. The list of STAT5b biomarker genes is found in S2 File. **List of genes in the STAT5b biomarker**.

### Functional analysis of STAT5b biomarker genes

The Comparative Toxicogenomics Database (CTD; http://ctdbase.org/) was used to find relationships that were annotated between chemicals and genes. Only the annotations “decreases^expression”, or “increases^expression” were used. A master table was built comprised of genes in the STAT5b biomarker and annotations from CTD using common gene abbreviations. The full list of genes in the STAT5b biomarker was analyzed using the IPA canonical pathway and upstream analysis functions. All results were exported as Excel files and filtered based on p-value and ratio.

### Comparison of STAT5b biomarker gene set to a microarray database

A rank-based nonparametric analysis strategy called the Running Fisher algorithm [34] and implemented within the NextBio database environment (http://www.nextbio.com/) was used for evaluating changes in STAT5b function. The algorithm is analogous to the Gene Set Enrichment Analysis (GSEA) method [43, 44]. As originally described by Lamb et al. (2006) [43], rank-based enrichment statistics allows a gene biomarker to be compared to large collections of high-throughput data to find associations with various factors including diseases and drug treatments. As a ranking metric, fold-change has been shown to have greater concordance than p-values from statistical tests as fold-change rank is platform-independent [45]. The Running Fisher algorithm computes p-values by a Fisher exact test (see Kuperschmidt et al. (2010) [34] for details), whereas GSEA uses permutations to assess statistical significance. This normalized ranking approach implemented by NextBio using the fold-change ranking enables comparability across data from different studies, platforms, and analysis methods by removing dependence on absolute values of fold-change, and minimizing some of the effects of normalization methods used, while accounting for the level of genomic coverage by the different platforms. Using the Running Fisher algorithm, the STAT5b biomarker gene set was compared to each bioset in NextBio. The p-values of the similarity and the correlation direction were exported, and after conversion of the p-values to—log(p-value) and negative correlations to negative numbers, were used to populate the table with study characteristics. This final master table enabled the determination of effects on STAT5b function by broad categories of factors (e.g., diet) as well as individual factors (e.g., fasting).

About ~400 biosets were derived by our methods and those used by NextBio. An analysis of the effects of procedures to derive the statistically significant genes is discussed in detail in S1 File. Given that the NextBio procedures are less stringent than those employed in our database due to the lack of a multiple test correction, it was not surprising that in general the NextBio method resulted in greater numbers of genes that overlapped with the STAT5b biomarker. However, despite this difference in numbers of overlapping genes, the predictions for STAT5b activation or suppression were strongly concordant between the two methods (R^2^ = 0.86). Based on these results, in subsequent analyses, the biosets derived by our methods were used in the analysis.

### Classification prediction of STAT5b function

Biosets from microarray experiments in which the STAT5b activation state was known were manually curated from the following studies: GSE55084, E-MEXP-1176, E-MEXP-1503, E-MEXP-153, E-MEXP-1711, E-MEXP-2209, E-MEXP-2539, E-MEXP-2636, E-MEXP-347, E-TABM-1139, E-TOXM-18, GSE10285, GSE10390, GSE10493, GSE11338, GSE1148, GSE11796, GSE13264, GSE13265, GSE13388, GSE14395, GSE15458, GSE17442, GSE17925, GSE19272, GSE20920, GSE21048, GSE21065, GSE24256, GSE24272, GSE29764, GSE30140, GSE31638, GSE32244, GSE35058, GSE6632, GSE7169, GSE7170. The number of biosets used to test for an increase in STAT5b function (i.e., STAT5b ‘activation’, associated with masculinization of liver gene expression) was 43 positives and 140 negatives. The number of biosets used to test for a decrease in STAT5b function (i.e., STAT5b ‘suppression’, associated with feminization) was 53 positives and 129 negatives. Unlike more traditional machine learning classification methods, optimal conditions for classification were not derived from gene behavior as the biomarker was fixed. In this and in our previous studies[31–33], the biomarkers were compared to known positives and negatives using the Running Fisher algorithm. Preliminary studies with the biomarkers for AhR, CAR and PPARα showed that a cutoff of Running Fisher algorithm p-value ≤ 10^−4^ (after a Benjamini Hochberg correction of α = 0.001) resulted in a balanced accuracy of 95%, 97%, and 98% for AhR, CAR and PPARα, respectively [31–33]. Applying the same test to the STAT5b dataset resulted in a cutoff of p-value = 2E-4 which for consistency with the previous studies was rounded to p-value = 1E-4. Use of a cutoff of 2E-4 vs. 1E-4 would have resulted in an additional 15 positives (4 masculinized and 11 feminized) out of the ~1850 biosets examined. Inclusion or exclusion of these biosets would not have altered any of the conclusions of the study. Using a cutoff of 1E-4 resulted in a balanced accuracy of STAT5b activation (masculinization) or STAT5b suppression (feminization) of 99% or 97%, respectively (Results and Discussion).

### Comparison of the expression of individual genes with STAT5b activation

Expression data for specific genes (i.e., *Igf1*, *Igfals*, *Igfbp3*) was obtained from the NextBio database based on microarray experiments. All statistically significant expression values (fold-changes) for each gene were used to populate the compendium, allowing a direct comparison between changes in specific genes and STAT5b predictions. In making the comparisons, only biosets in which gene expression was altered were used in the comparisons, as values of “0” may also represent “no value” if the microarray platform did not query the gene or allow assessment of changes in that gene.

### Additional computational analyses

Principal component analysis (PCA) was evaluated using unfiltered fold-change values of the STAT5b biomarker genes from biosets examined by Affymetrix 430_2 or 430A chips using Eisen Lab Cluster (http://rana.lbl.gov/EisenSoftware.htm) and visualized by SigmaPlot. Heat maps were generated using Eisen Lab Cluster and Treeview software (http://rana.lbl.gov/EisenSoftware.htm).

## Results

### Construction and characterization of a STAT5b gene expression biomarker

**Fig 2:**
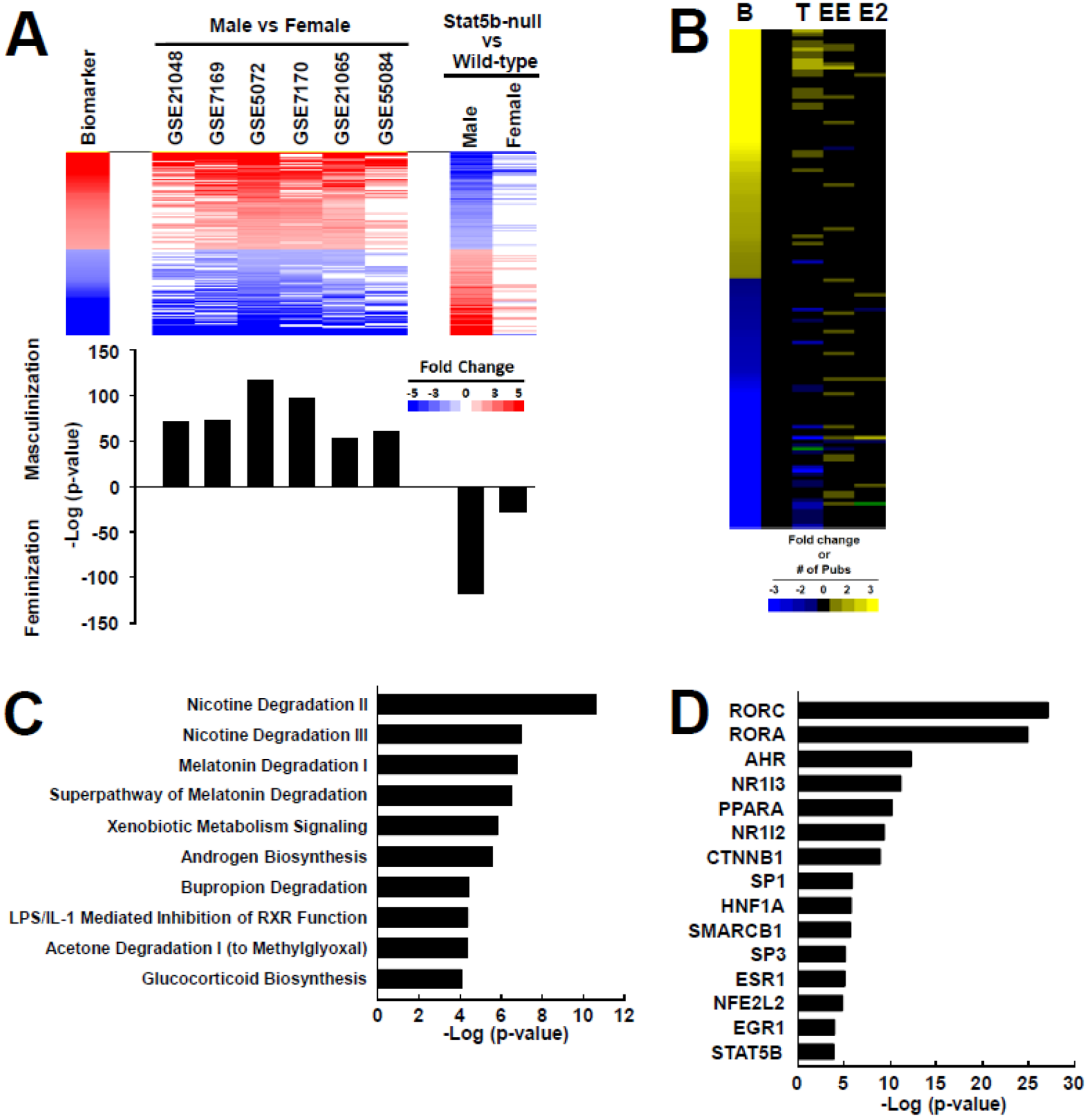
Characterization of a STAT5b biomarker. A. Comparison of the STAT5b biomarker gene set to biosets used to create the biomarker. (Top) Gene expression profiles from six male to female comparisons from the indicated experiments (GEO numbers) were used to identify genes with similar behavior across the comparisons. Genes lists were filtered to identify liver-expressed genes that were regulated by STAT5b based on comparisons between livers of STAT5b-null and wild-type mice, in both males and females [16]. The biomarker represents the average expression of the genes across the male to female comparisons. The genes were rank-ordered based on biomarker gene expression. (Bottom) The gene biomarker was compared to the biosets using the Running Fisher algorithm. Biosets with a negative correlation are shown as negative—log(p-value)s. B. Expression behavior of the STAT5b biomarker genes in the CTD. Information regarding the direction of change of the STAT5b biomarker genes (B) annotated in CTD for treatment of mice with testosterone (T), ethinyl estradiol (EE) and estradiol (E2) is shown. For the biomarker genes, expression is shown as increased (yellow) or decreased (blue) in fold-change. In the T, EE and E2 columns, the intensity of the each gene reflects the number of publications in which the expression of that gene is altered. If two or more publications indicate both increased and decreased expression, then the gene is represented as green. Black represents no information. Inconsistencies in expression changes with the biomarker may be attributed to effects observed in tissues other than the liver, as CTD does not document the tissue origin of the change. C. Top canonical pathways represented by the genes in the STAT5b biomarker. Genes were examined by Ingenuity Pathways Analysis. D. Transcription factors predicted to act as regulators of STAT5b biomarker genes, as determined by Ingenuity Pathways Analysis.

STAT5b biomarker genes were identified using microarray profiles from the livers of wild-type male and female mice based on 6 individual experiments carried out in 4 different labs (see Methods). Genes showing consistent male-female differences in expression across the 6 studies were further filtered for regulation by STAT5b, as indicated by significant expression differences between livers of male STAT5b-null mice and male wild-type mice [16]. A total of 144 genes (74 with increased expression and 70 with decreased expression) showed similar regulation in at least 4 out of the 6 male to female comparisons. Further, all genes in this biomarker gene set were dependent on STAT5b for their sexual dimorphic expression (Fig 2A, **top**). Genes that exhibited greater expression in livers of wild-type male mice than in wild-type female mice were decreased in the absence of STAT5b, and genes that exhibited lower expression in wild-type male mice than in wild-type female mice were increased. This pattern is consistent with well-established roles of STAT5b in both activation and suppression of sex-dependent genes in male mouse liver[16]. Gene expression changes were far less dramatic in female STAT5b-null mice, consistent with the lesser role of STAT5b in regulating sex-biased genes in female compared to male liver[16]. The 144 STAT5b biomarker genes (S2 File. **List of genes in the STAT5b biomarker**) fall into a number of subclasses that differ in their responses to changes in GH and regulation by the transcription factors STAT5b and Hnf4a (reviewed in Waxman and Holloway, 2009 [11].

To determine whether genes in the STAT5b biomarker set are regulated by androgen or estrogen receptor activators in other published studies, we used the Comparative Toxicogenomics Database (CTD; http://ctdbase.org/) to find annotated relationships between hormone exposure and expression of the biomarker genes. Gene-hormone interactions for testosterone, ethinyl estradiol, and estradiol are shown in Fig 2B for the 136 genes annotated in CTD that overlap with the STAT5b biomarker set. Many of the gene-hormone interactions for the genes in the biomarker were annotated for testosterone, and these genes exhibited directional changes after testosterone exposure (i.e., increases or decreases in expression) consistent with the changes of the genes in the biomarker set. Far fewer interactions were annotated for ethinyl estradiol and estradiol, and in general, there was no apparent relationship between estrogen exposure and expression of the STAT5b biomarker genes, indicating the biomarker genes are not under estrogen control. While there is evidence of effects of estrogens on STAT5b-dependent *Cyp* gene expression (discussed in detail in our companion paper [30]), it should be noted that CTD does not annotate the tissue for the chemical (hormone)–gene interaction and thus, some (perhaps many) of these interactions may occur in tissues other than liver.

The STAT5b biomarker genes were evaluated for canonical pathway enrichment by Ingenuity Pathway Analysis (IPA) (Fig 2C). The top 10 pathways enriched with the biomarker genes included many associated with STAT5b gene targets, including Androgen Biosynthesis and Xenobiotic Metabolism Signaling as well as pathways related to xenobiotic metabolism (nicotine, melatonin, bupropion, and acetone degradation). Indeed, the STAT5b biomarker set includes 37 genes with functions in xenobiotic metabolism or transport, including phase I xenobiotic metabolism genes well known to be regulated in a sex-dependent manner (e.g. *Cyp2d9*, *Cyp7b1*, and *Cyp2b13)*. The biomarker genes involved in testosterone metabolism are apparently regulated by a feed-forward mechanism that increases availability of testosterone or the more potent dihydrotestosterone. Thus, genes involved in steroid synthesis (*Hsd3b1*, *Hsd3b4*) as well as testosterone 5α-reductase (*Srd5a1*), which converts testosterone to dihydrotestosterone, exhibited increased expression in the biomarker, while decreased expression in the biomarker set was seen for two out of the three genes active in testosterone 16α-hydroxylation (*Cyp2b13* and *Cyp2b9* but not *Cyp2d9*), which decreases the activity of testosterone. Other genes in the biomarker set, including genes involved in xenobiotic metabolism, are targets of STAT5b, and underlie sexual dimorphic responses to xenobiotic exposure [16]. Although the enrichment of genes in the pathway of Glucocorticoid Biosynthesis (Fig 2C) is intriguing given the known interactions between STAT5b and glucocorticoid receptor [46], all of those genes are also included in the set of Androgen Biosynthesis genes discussed above.

The upstream analysis function of IPA identified a number of transcription factors that are predicted to regulate the STAT5b biomarker genes (Fig 2D). The top 15 scoring transcription factors included nuclear receptors, i.e., retinoid activated receptor-related orphan receptors (RORA, RORC), PPARα, CAR (Nr1i3), and PXR (Nr1i2), as well as STAT5b itself. Additionally, Ahr, Nfe2l2 (Nrf2), and HNF1a were predicted to be upstream regulators, possibly because of the over-representation of xenobiotic metabolism genes regulated by these transcription factors. These findings are remarkably similar to those recently described for a common set of genes regulated by *Stat5b*, *Ghrhr* (Growth hormone-releasing hormone receptor), and *Androgen receptor* [47]. In summary, the STAT5b biomarker includes genes with the expected activities, including xenobiotic metabolism and testosterone biosynthesis and metabolism.

### A rank-based strategy to predict STAT5b activation

A rank-based analysis called the Running Fisher algorithm [34] was used to predict activation or suppression of STAT5b function in individual gene biosets in the mouse liver compendium. These biosets are lists of genes that exhibit statistically significant changes in expression between two states, e.g., chemically-treated vs. control treated or nullizygous gene vs. wild-type gene. In previous studies, the Running Fisher algorithm coupled with derived gene biomarkers was found to be very accurate (balanced accuracy range: 95%–98%) in predicting the activation of xenobiotic-responsive transcription factors AhR, CAR and PPARα using the same mouse liver compendium queried in the present study [31–33]. First, we used the Running Fisher algorithm to compare the STAT5b biomarker gene set to the 6 male vs. female biosets used to create the STAT5b biomarker set. As expected, all 6 biosets exhibited highly significant similarity to the STAT5b biomarker (p-values ≤ 10^−53^). Biosets derived from male and female STAT5b-null vs. wild-type mice[16] exhibited a highly significant negative correlation to the biomarker, as expected (Fig 2A, **bottom**).

**Fig 3:**
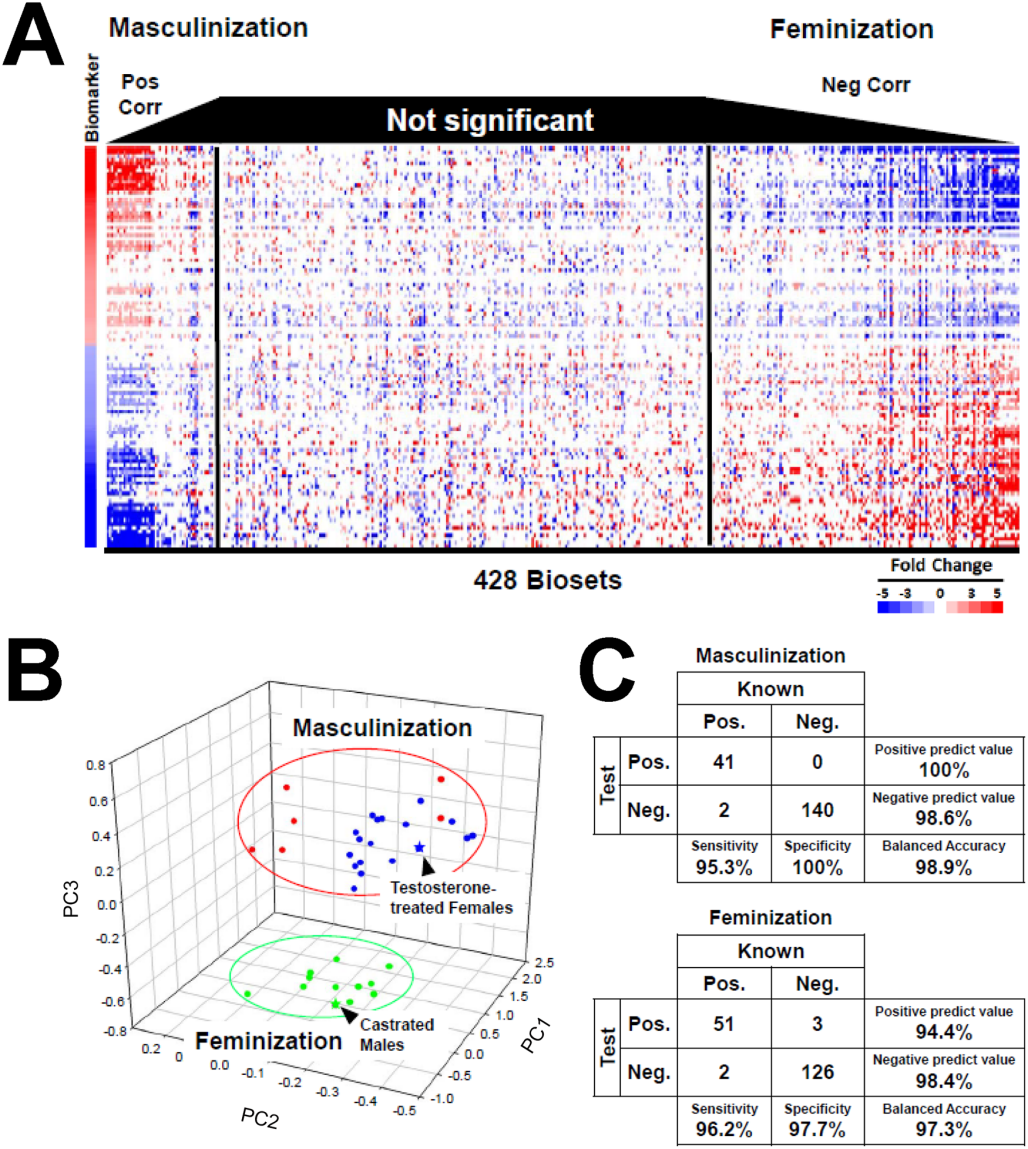
Rank-based strategy for prediction of STAT5b activation status. A. Heat map showing the expression of genes in the STAT5b biomarker across 428 biosets. Biosets were ordered based on their similarity to the STAT5b biomarker using the—log(p-value) of the Running Fisher test. Biosets with positive correlation (masculinization) are on the *left* and biosets with negative correlation (feminization) are on the *right*. Black vertical lines denote the p-value = 10^−4^ cutoff used in the study. B. The biomarker genes separate known positives and negatives for liver STAT5b activation using principal components analysis. The first three principal components are shown, derived from the unfiltered expression changes of the biomarker genes across the biosets. Red, the 6 male to female comparisons used to create the STAT5b biomarker. Blue, 17 male to female comparisons from wild-type adult mice. Green, 11 female to male comparisons from wild-type adult mice. Blue star, comparison between female mice treated with 0.9 mg/kg dihydrotestosterone (DHT) by subcutaneous injection 2 times a week for 3 weeks vs. control (GSE13388). Green star, castrated vs. intact male mice (GSE6632). C. Summary of the sensitivity and specificity of the STAT5b biomarker. The biomarker was compared to biosets that were known positives or negatives for STAT5b activation. Separate tests for STAT5b activation (masculinization) and STAT5b suppression (feminization) were carried out.

The STAT5b biomarker was next compared to the large collection of biosets comprising the mouse liver gene expression compendium. Biosets showing strong similarity to the STAT5b biomarker gene set, indicated by a low p-value from the Running Fisher test, are expected to exhibit a pattern of gene expression similar to that of the biomarker. To visualize the relationship between gene expression in the biomarker and the Running Fisher algorithm p-value, 428 biosets, each comprised of statistically-filtered genes, were compared to the STAT5b biomarker and sorted by p-value. Fig 3A **(left)** shows that for biosets with a positive correlation to the biomarker, the lower the p-value, the greater the similarity between the bioset and the biomarker gene set in both the direction and the magnitude of the gene expression changes. These biosets include many comparisons between male and female mice, including two of the comparisons used to create the biomarker set (described in greater detail below). Fig 3A **(right)** shows biosets that exhibited a negative correlation to the biomarker, with the biosets on the far right exhibiting the lowest p-values for negative correlation. Several of these biosets compare female vs. male mice, or male wild-type mice vs. male mice deficient in genes that regulate GH responses (e.g., *Ghr*, *Pit1 (Pou1f1)*, *Prop1*, *Ghrhr* (discussed below)) and exhibited patterns of gene expression opposite to that of the STAT5b biomarker. We therefore describe biosets that have a significant positive correlation to the STAT5b biomarker as exhibiting masculinization of the liver; biosets that exhibit a significant negative correlation to the STAT5b bioset exhibit feminization.

The STAT5b biomarker was evaluated for its ability to correctly classify biosets as those that exhibit masculinization or feminization. Biosets of known positives and negatives were first examined by principal components analysis (PCA) to determine if the expression behavior of the genes in the STAT5b biomarker can separate the biosets into two groups (masculinized vs. feminized liver gene expression). PCA was evaluated using unfiltered fold-change values of the STAT5b biomarker genes examined on Affymetrix 430_2 or 430A gene chips. Fig 3B shows a clear separation of biosets with known masculinization (blue and red dots within the red oval) from biosets with known feminization (green dots within the green oval). Examples include a bioset of testosterone-treated female vs. vehicle-treated female liver (**blue star**), which segregates to the masculinization group, and a bioset of male liver 9 months after mouse castration vs. intact males, which segregates to the feminization group (**green star**) (Fig 3B).

A classification was performed using a large number of biosets in which the STAT5b activation state was known. For prediction of masculinization, the STAT5b biomarker set had a 95% sensitivity and a 100% specificity, with a balanced accuracy of 99% (Fig 3C, **top**). For prediction of feminization, the STAT5b biomarker set had a 96% sensitivity and a 98% specificity, giving a balanced accuracy of 97% (Fig 3C, **bottom**). Thus, the STAT5b biomarker has an excellent balanced accuracy to detect masculinization or feminization in biosets from the compendium.

### Analysis of a mouse liver gene expression compendium

The STAT5b biomarker and the Running Fisher algorithm were used to identify biosets in which liver STAT5b function was altered. The compendium of biosets examined consisted of gene expression changes in mouse livers, mouse primary hepatocytes, and mouse hepatocyte-derived cell lines. The compendium contains ~1850 biosets of gene expression changes between control and experimental states, including ~470 chemicals, ~450 genes, ~220 dietary conditions, ~100 hormones or cytokines, ~90 life stage, ~90 stress and ~120 strain comparisons.

**Fig 4:**
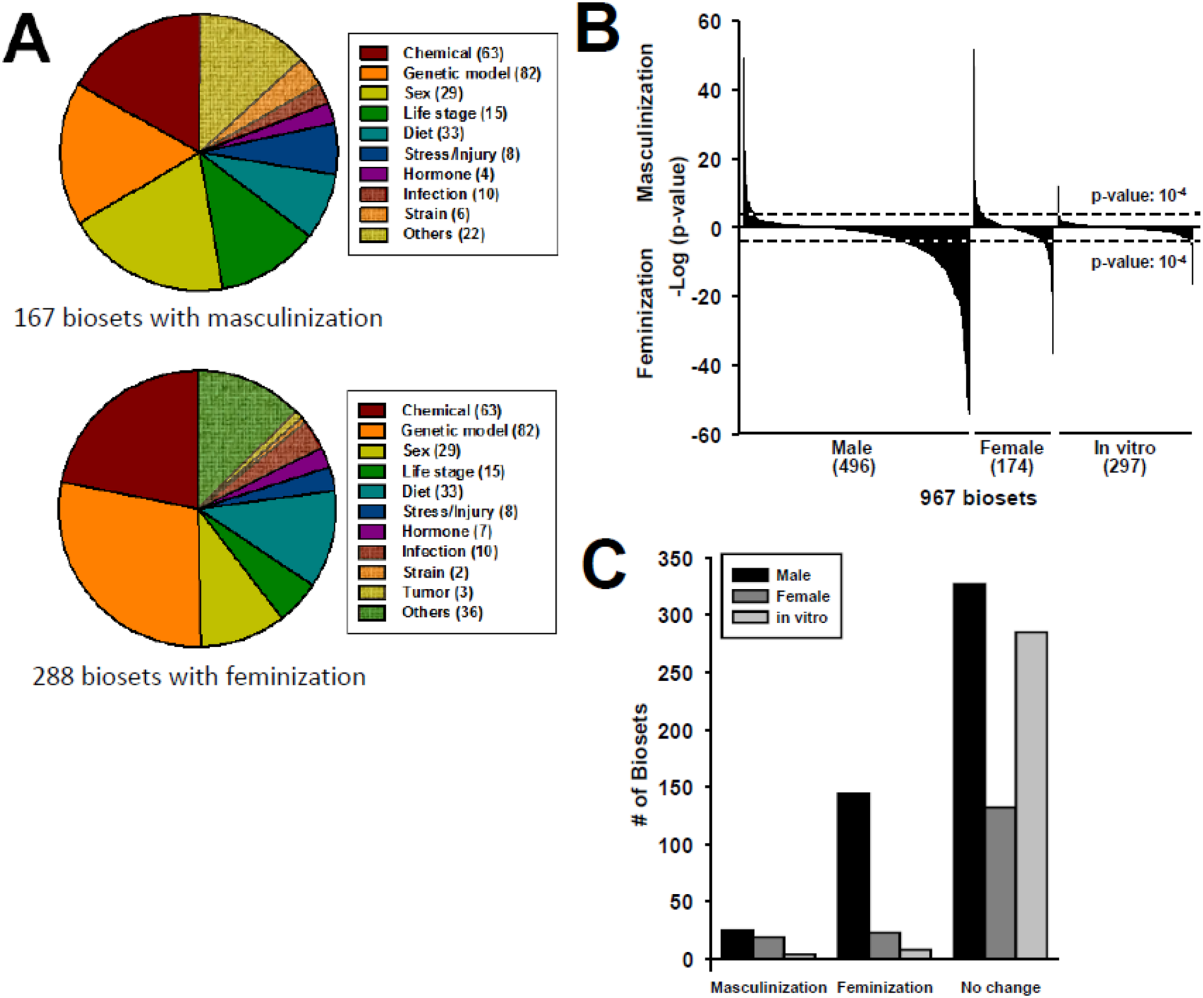
Assessment of STAT5b function in a mouse liver compendium. A. Assessment of factors that alter STAT5b function, leading to activation (masculinization) or suppression (feminization) across biosets in a mouse liver compendium. The STAT5b biomarker was compared to ~1850 biosets using the Running Fisher test. The number of biosets with a p-value ≤ 10^−4^ for either activation or suppression in the indicated categories are shown. B. Distribution of—log(p-value)s for all biosets derived from male mice, female mice or in vitro experiments. The p-value cutoffs are shown. C. Number of biosets that exhibit masculinization or feminization in intact male and female mice and in in vitro experiments.

The factors that affect STAT5b function are summarized in Fig 4A. Of the factors examined, genotypic sex (i.e., male vs. female, or female vs. male comparisons), chemicals, and genetic models have the greatest number of effects on STAT5b function. To determine the relationships between sex of the experimental model and effect of the different factors on the animals, biosets were divided into those from male mouse liver (496 biosets), female mouse liver (174 biosets), and those based on in vitro studies using hepatocytes or hepatocyte-derived cell lines from either sex (297 biosets). Surprisingly, many of the experiment descriptions in GEO and even in the original papers did not specify the sex of the mice used in the experiments (discussed in the Methods).

Male mice appeared to be particularly sensitive to feminization, with ~29% of the male biosets feminized by the treatments investigated, while ~5% were masculinized (Fig 4B and 4C). In females, biosets were almost equally distributed between those that were masculinized (~11%) and those that were feminized (~13%). Many of the effects of factors tested on feminization were more significant in males (p-values ≥ 10^−55^) than effects of feminization in females (p-values ≥ 10^−35^). Because in vitro cultures generally lack the ability to respond normally to the male-dependent pattern of pulsatile GH secretion (Waxman and Holloway, 2009), we predicted that in vitro liver cell cultures would not exhibit significant changes in STAT5b function. Indeed, only a total of 11 (4%) of the biosets from in vitro cultures reached significance (4 masculinized and 7 feminized), and the range of significance of these correlations (p-values ≥ 10^−7^) was far less than the range of significance values for the in vivo comparisons (p-values ≥ 10^−55^).

The masculinization of male mice and feminization of female mice requires an explanation, given the expectation that adult males are already masculinized and adult females are already feminized in the absence of perturbation. The observed masculinization in a small subset (~5%) of male samples can be explained for a small number of biosets if the “controls” represent a physiological state in which the males were previously feminized. Examples of this are presented in our companion study [30], and include castrated male mice (which are feminized) administered dihydrotestosterone, resulting in masculinization. However, for the majority of cases the masculinization is observed when compared to untreated intact males (e.g., nullizygous mutants of *Ppara* or *Nr2c2* discussed below). Similarly, feminization of female mice could occur if the “controls” already exhibit a more masculine state that is perturbed by the factor in the direction of feminization, but there are no clear examples of this in the feminized biosets from female mice. An examination of the gene expression pattern of the biomarker genes in female mice that exhibited feminization showed that the overall patterns of change do not differ from those seen in male mice that are feminized, i.e., male-biased genes are down-regulated and female-biased genes are up-regulated (data not shown). The physiological and biochemical explanations for these feminization responses have not been established in most cases, but may include alteration in the female pattern of continuous GH secretion.

In summary, feminization of the male-specific liver gene expression pattern was a frequent event, occurring in ~29% of all the biosets from male mice. Disruption of the male-specific pattern of liver gene expression is thus a relatively common occurrence. This disruption may be due to multiple factors, including perturbation of one or more nodes on the HPL axis, as well as the sensitivity of the male-specific pattern of pituitary GH secretion to feedback inhibitory mechanisms. Overall, STAT5b function was altered in ~34% and ~14% of all biosets examined in male and female mouse liver, respectively. This can be compared to 8%, 27%, and 14% of the biosets from the same mouse liver gene expression compendium exhibiting either activation or suppression of liver AhR, CAR or PPARα function, respectively [31–33].

### Life stage-dependent alterations of STAT5b function

**Fig 5:**
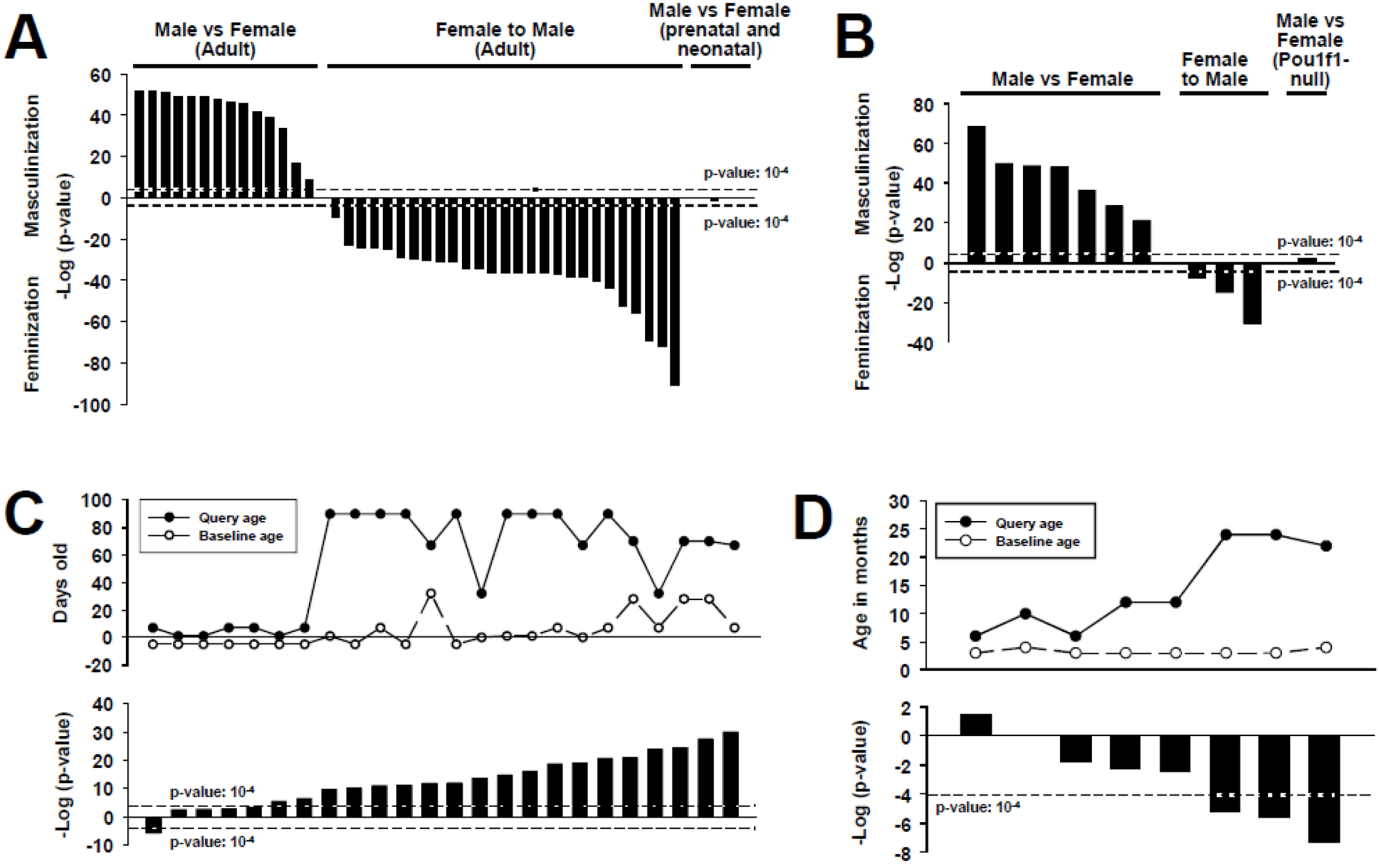
Life stage-dependent alterations in mouse liver STAT5b function. A. Comparison of the STAT5b biomarker gene set to sex-based comparisons from wild-type mice. The STAT5b biomarker was compared to adult male vs. female biosets (*left*), adult female vs. male biosets (*middle*), and male vs. female biosets from prenatal (E15.5) and postnatal (PND2 and PND14) mice (*right*). Dashed horizontal lines indicates the threshold for significance (p-value ≤ 10^−4^). B. Comparison of the STAT5b biomarker gene set to male vs. female (*left*), female vs. male (*middle*) biosets from nullizygous mouse models that retain sexual dimorphic gene expression behavior, or a male vs. female comparison from a dwarf mouse model lacking normal GH secretion (*Pou1f1*). C. STAT5b activation during sexual maturation in male mice. (Top) Query and baseline ages (in days postnatal) of the male mice used in each comparison. (Bottom) Significance of the similarity between the STAT5b biomarker gene set and each of the biosets. D. Feminization of the male liver transcriptome in aging mice. The STAT5b biomarker gene set was compared to biosets from male mice evaluating the effects of age on gene expression. (Top) Query and baseline ages (in months) of the mice used in the comparisons. (Bottom) Significance of the similarity between the STAT5b biomarker and the biosets.

The STAT5b biomarker gene set was used to examine relationships between hepatic STAT5b activity and life stage of the test animals. As expected, adult male vs. female comparisons from wild-type mice gave significant positive correlations (p-values = 10^−7^ to 10^−77^) (Fig 5A **(Left)**), whereas female vs. male comparisons gave significant negative correlations (p-values = 10^−9^ to 10^−54^) (Fig 5A **(Middle)**). Given that sex-specific gene expression is not manifested in mouse liver until after ~3–4 weeks after birth [48, 49], we examined male vs. female liver comparisons in prenatal and neonatal mice, ranging from gestation day (GD) 15.5 to postnatal day (PND) 2 and PND14. None of these biosets exhibited significant correlations, as was anticipated (Fig 5A **(Right)**). Examination of biosets comparing adult male vs. female mice or adult female vs. male mice in various nullizygous mouse models showed that most of the models exhibited significant positive or negative correlations, for comparisons of male vs. female, or female vs. male, respectively (Fig 5B). Notably, one of the male vs. female comparisons that showed a significant correlation was derived from STAT5a-null mice, supporting the earlier conclusion that this transcription factor, which is expressed in liver at a much lower level than STAT5b, and whose protein sequence is >90% similar to that of STAT5b, is not essential for male-specific liver gene expression [16]. In contrast, significant correlations with the STAT5b biomarker gene set were abolished in a male vs. female comparison from mice containing a mutation in *Pit1/Pou1f1*, which encodes a transcription factor required for development of the anterior pituitary and GH secretion (bioset E-MEXP-347).

There are two periods of rapid growth in mice: the first is perinatal and independent of GH and the second is peripubertal and GH-dependent. STAT5b is the primary transcription factor that GH activates to induce insulin-like growth factor 1 (IGF1) synthesis and somatic growth [48]. The ability of the STAT5b biomarker to identify peripubertal masculinization in male mice was examined. Biosets in the compendium from the livers of male mice aged PND6 to PND95 compared to mice GD14.5 to PND35 came from 3 studies (GSE10785, GSE16675, GSE21224). Only the biosets from mice that reached sexual maturity (ages ranging from PND32 to PND90) compared to prenatal or postnatal mice (ages ranging from GD14.5 to PND28) were positively correlated to the STAT5b biomarker (p-values = 10^−11^ to 10^−36^) (Fig 5C). These results are consistent with low levels of (active) nuclear phosphorylated liver STAT5 in one-week animals and greater levels in 2.5-week animals similar to adult 9-week controls in the mouse model, coinciding with the onset of the GH-dependent phase of growth[48], and with a similar pattern of increased liver STAT5 DNA binding activity in pubertal and adult compared to prepubertal rat liver [50]. Most of the biosets from postnatal mice (PND1 or PND7) compared to prenatal mice (GD14.5) were not significantly correlated. However, the feminization of the male liver transcriptome at PND7 (when compared to GD19) seen in one bioset was described previously [51].

GH secretion levels decline with age, and sexually dimorphic differences in gene expression become less evident in older males [29]. In mice, these changes are likely driven by decreases in circulating testosterone observed in aging males [52]. Consistent with this phenomenon, biosets from old wild-type male mice (22 or 24 months) compared to younger counterparts (3 or 4 months) showed significant feminization (Fig 5D). Overall, the behavior of the STAT5b biomarker gene set is consistent with known effects of different life stages on liver STAT5b activity, including masculinization during the perinatal period of growth in males and feminization in aging males.

### Effects of diets and infections on STAT5b

Effects of diets and infections on STAT5b are discussed in S1 File.

### Relationships between STAT5b and gene expression of components of bioactive IGF-1

The relationships between STAT5b and expression of components of bioactive IGF-I are discussed in S1 File.

### Identification of genes that alter hepatic STAT5b function

**Fig 6:**
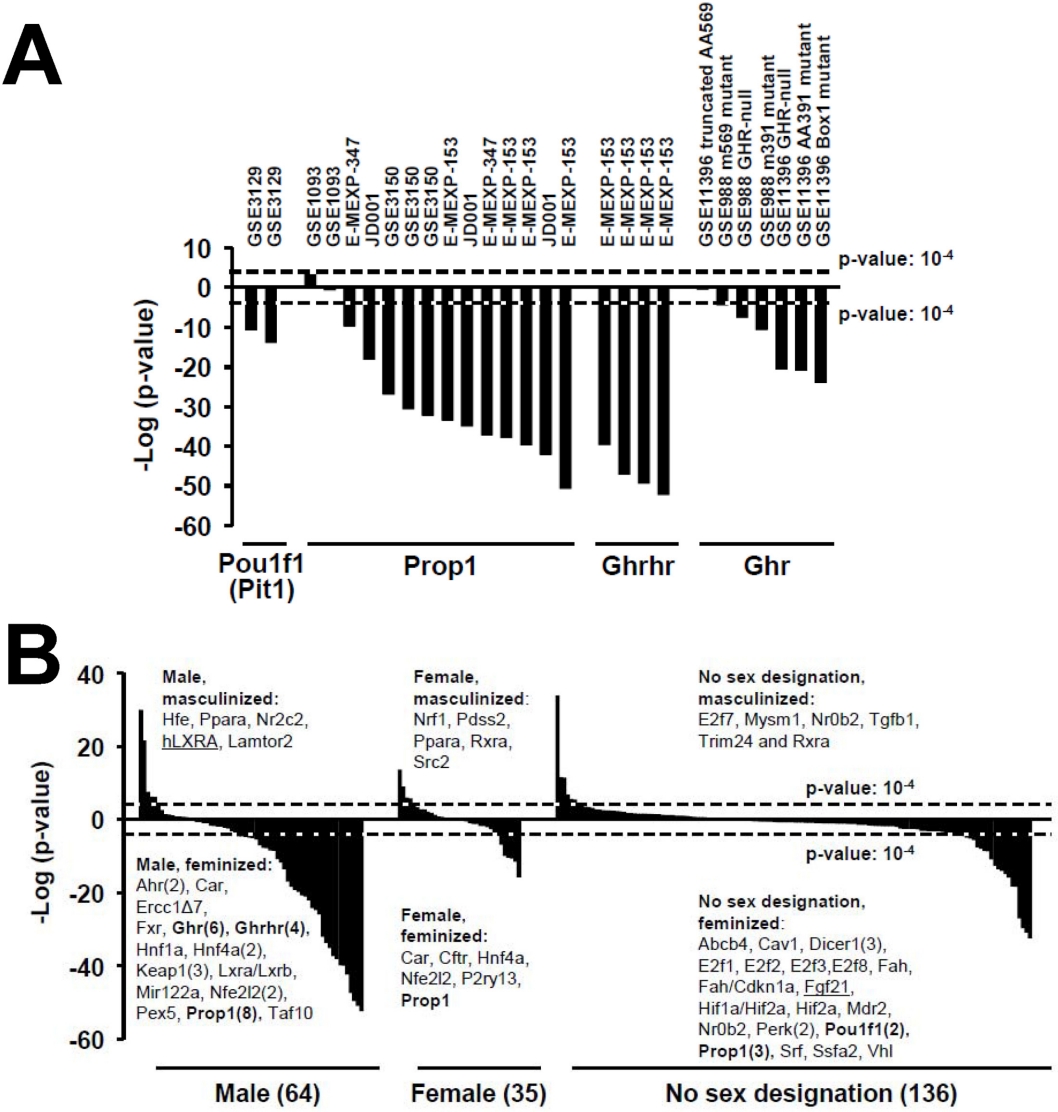
Genetic activation or suppression of STAT5b function. A. Inactivation of genes involved in GH secretion or GH signaling results in feminization of the liver transcriptome. Biosets from the livers of mice with inactivated genes (*Pit1 (Pou1f1*, *Prop1*, *Ghrhr* and *Ghr*) compared to their wild-type controls almost universally exhibited negative correlation (feminization) to the STAT5b biomarker gene set. B. Masculinization or feminization in genetic models. The gene expression profiles derived from the indicated mutant mice vs. wild-type controls were compared to the STAT5b biomarker gene set. The effects were divided into those from male mice, female mice, and mice with no annotation for sex (see Methods). Underlined genes indicate mouse models in which the gene is over-expressed; all others are models in which the genes have been knocked down or knocked out. Masculinization was observed in mice that were nullizygous for both *Trim24* and *Rxra*. Genes in bold are those that affect GH secretion or signaling shown in A. Human genes that have been knocked in are indicated with an “h” before the gene name. Numbers in parentheses indicate the number of biosets that examined effects of that gene if more than one bioset was examined.

Genetic changes that impact liver STAT5b function were examined. Mutations in genes that affect GH secretion or activity were examined first; these included biosets from mouse models with mutations that 1) abolish GH secretion because of defects in the differentiation of the anterior pituitary (*Pou1f1 (Pit1)*, *Prop1*), 2) suppress the synthesis and secretion of GH by the pituitary (*Ghrhr*), or 3) affect the function of GH receptor (*Ghr*). All of these mutations result in dwarfism [53]. Fig 6A shows that most of these biosets result in significant suppression of liver STAT5b function (feminization), as expected. Only two of the 14 *Prop1* comparisons were not associated with significant feminization (both from one study, GSE1093). Biosets characterizing GH receptor truncated at amino acid 569 (GSE11396, GSE988), were either not significant or were marginally significant for feminization (p-value = 0.33 and 3.8E-05, respectively), consistent with the weak to moderate effects of this mutant on GH-dependent phosphorylation of STAT5b [54]. In contrast, GH receptor truncation at amino acid 391 or deletion of the GH receptor Box1 domain (the JAK2 binding site) abolished STAT5b phosphorylation [55], and in our study resulted in significant feminization (p-values = 10^−7^ to 10^−24^). Thus, the STAT5b biomarker gene set accurately identified mouse mutants in which GH secretion or activity was impaired.

The effects of gene inactivation or overexpression on mouse liver STAT5b function were examined in 235 biosets encompassing 125 mouse genes (Fig 6B). The biosets were divided into those with effects in male mice, female mice or in mice where no information about sex was provided. Most of the genetic changes were associated with feminization of the male liver transcriptome. Genes that when nullizygous led to feminization in males included genes with direct effects on GH regulation discussed above (shown in bold in Fig 6B), and genes encoding nuclear receptors (*Car*, *Fxr*, *Hnf4a*, *Lxra/Lxrb*), other transcription factors (*Ahr*, *Hnf1a*, *Taf10*), Nrf2 signaling components (*Keap1*, *Nfe2l2*), and miscellaneous genes (*Ercc1Δ7*, *Mir122a*, *Pex5*). Masculinization in male mice was seen in mice nullizygous for nuclear receptors *Nr2c2* (also called *Tak1/TR4)* and *Ppara*, as well as *Hfe* and *Lamtor2*, and in mice overexpressing human *LXRA*.

Compared to males, fewer effects were observed in female mice. Examples included genes that when inactivated caused masculinization (*Nrf1*, *Pdss2*, *Ppara*, *Rxra*, *Src2*) or feminization (*Car*, *Cftr*, *Hnf4a*, *Nfe2l2*, *P2ry13*). In studies that did not provide information about the sex of the animals, several additional genes were identified that when nullizygous either caused masculinization (*E2f7*, *Mysm1*, *Tgfb1*, *Trim24* and *Rxra* double nullizygous) or feminization (*Abcb4*, *Cav1*, *Dicer1*, *E2f1/E2f2/E2f3*, *E2f8*, *Fah*, *Cdkn1a*, *Mdr2*, *Nr0b2*, *Perk*, *Srf*, *Ssfa2*, *Vhl*). Overexpression of *Fgf21*, both *Hif1a* and *Hif2a*, or *Hif2a* alone caused feminization.

Several of the genes identified here as having effects on liver STAT5b function can interact with GH signaling pathways; some of these genes act as negative regulators of GH signaling in intact mice and when inactivated cause masculinization of the liver, as follows.

- NR2C2 (nuclear receptor subfamily 2, group C, member 2) is also known as testicular receptor 4, TR4, or TAK1. Tak1-null mice demonstrate high rates of early postnatal mortality, as well as significant growth retardation [56]. Serum levels of IGF-1 are reduced by 34% in Tak1-null mice compared with wild-type controls; however, GH levels were normal in the anterior pituitary [57], and effects of Tak-1 knockout on the pulsatility of pituitary GH secretion was not investigated. The low serum IGF-1 levels and reduced IGF-1 immunoreactivity in Tak1-null mouse liver indicates that an IGF-1 deficiency may contribute to the growth retardation observed [57]. One year-old Tak1-null mice were protected from obesity-linked inflammation, hepatic steatosis, and insulin resistance [56]. In our companion study [30], feminization is often observed in mice that are genetically- or diet-induced obese and/or diabetic, and thus the masculinization seen in Tak1-null mice could be due to resistance to the feminization effects of obesity/diabetes that normally occurs in one year old wild-type mice.
- Trim24 (tripartite motif-containing 24) is a transcription factor expressed in adult female rat and mouse liver at levels up to 73-fold higher than in male counterparts [58]. Liver expression of *Trim24* is regulated by CUX2, a highly female-specific liver transcription factor [59], which itself is regulated by GH and dependent on STAT5b [58]. In our study, masculinization was observed in the livers of 5 wk old mice that were Trim24-null and Rara heterozygotes compared to wild-type mice. The masculinization approached significance (p-value = 4E-4) in 5 wk old Trim24-null mice vs. wild-type mice from the same study (GSE19675).
- TGF-β1 can be a negative regulator of STAT5b, as mast cells exhibited significantly reduced expression of *STAT5* upon TGF-β1 exposure. STAT5b overexpression blocked TGF-β1-mediated suppression of IgE-induced cytokine production [60].
- The masculinization caused by null mutations in *Ppara* or its heterodimeric binding partner *Rxra* in males and females (Fig 6B) is consistent with the mutually antagonistic interactions between PPARα and STAT5b [28]. In contrast to the masculinization effects of PPARα inactivation, feminization was observed after exposure to PPARα activators [32].
- Two genes that regulate gene expression through epigenetic mechanisms caused masculinization when inactivated. *Mysm1* regulates transcription by coordinating histone acetylation and deubiquitination, and destabilizing the association of linker histone H1 with nucleosomes [61]. Mysm1 participates in transcriptional regulation events in androgen receptor-dependent gene activation [61]. Src2/NCoA-2 is a transcriptional coregulator that contains several nuclear receptor interacting domains and an intrinsic histone acetyltransferase activity. Src2 in turn acetylates histones, which renders downstream DNA more accessible to transcription. GH actions induce rapid and dramatic changes in hepatic chromatin, including histone acetylation, at target promoters[62]. Given that STAT5b activity can be modulated through acetylation (reviewed in Zhuang, 2013[63]), we hypothesize that loss of *Mysm1* or *Src2* leads to loss of negative interference of liver STAT5b activity.
- Nr0b2, also known as small heterodimer partner (SHP), is a member of the nuclear receptor family and is unusual in that it lacks a DNA binding domain. Inactivation of *Shp* was found to both masculinize mice fed a western diet and feminize mice fed a normal diet. Shp negatively regulates GH stimulation of hepatic gluconeogenesis through inhibition of STAT5 transactivation [64]. Immunoprecipitation studies showed that SHP physically interacts with STAT5 and inhibits STAT5 recruitment to the *PEPCK* gene promoter. The increase in hepatic gluconeogenesis following GH treatment is significantly higher in livers of Shp-null mice compared with that of wild-type mice [64].

Genes that when inactivated cause feminization may encode positive regulatory factors for STAT5b activation in intact mice or cause loss of signaling of the HPL-GH axis.

- *Cav1* encodes a scaffolding protein that is the main component of the caveolae plasma membranes found in most cell types. Cav1 may offer resistance to *K*. *pneumoniae* infection, by affecting both systemic and local production of proinflammatory cytokines via the actions of STAT5 and the GSK3β-catenin-Akt pathway [65].
- Dicer1-null mice exhibited feminization in three biosets from two studies. *Dicer1* is critical for the biogenesis of mature microRNAs, which are short non-coding RNAs that regulate gene and protein expression. In one of the studies (GSE11899), hepatocyte-specific Dicer1-deletion and the associated deficiency in miRNA production leads to hepatocyte apoptosis, an increase in hepatocyte regeneration and portal inflammation, and derepression of many miRNA targets, most notably imprinted genes [66]. Although there is no information about a specific role of Dicer1 in regulating liver GH signaling or STAT5b activity, pituitary-specific Dicer1-null mice exhibited decreased expression of GH and other hormones, and decreased expression of *Pou1f1 (Pit1)*, involved in the differentiation of the anterior pituitary [67].
- *Cdkn1a* (cyclin-dependent kinase inhibitor 1a) inactivation resulted in feminization. Although effects of *Cdkn1a* on STAT5b signaling have not been previously observed, STAT5b was shown to negatively regulate cell proliferation through the activation of *Cdkn2b* and *Cdkn1a* expression [68]. We hypothesize that STAT5b and Cdkn1a cross-talk with one another.
- Serum response factor (SRF) is a transcription factor that binds to a CarG box motif within the serum response element of genes induced by mitogen stimulation. Compared to control mice, hepatocyte-specific Srf-null mice had lower blood glucose and triglyceride levels, were smaller, had severely depressed levels of serum IGF-1 as well as other components of GH/IGF-1 pathway [69], indicating defects in GH signaling.
- Ercc1Δ7 mutation was found to decrease the expression of genes in the GH/prolactin/thyrotrope axes [70] and to exhibit feminization [71].
- Overexpression of mouse fibroblast growth factor-21 (FGF21) caused feminization. FGF21 is a hormone secreted by the liver during fasting that elicits diverse aspects of the adaptive starvation response. Transgenic FGF21 mice exhibit reduced levels of STAT5b and corresponding decreases in the expression of STAT5b target genes, including IGF-1 [72]. FGF21 also induces hepatic expression of IGF-1 binding protein 1 and suppressor of cytokine signaling 2, which blunt GH signaling. Chronic exposure to FGF21 markedly inhibits growth [72] and markedly extends lifespan in mice by primarily blunting the GH/IGF-I signaling pathway in liver [18, 20]. Thus, mice that overexpress FGF21 exhibit phenotypes similar to mouse GH signaling mutants.

Feminization of liver STAT5b function was also associated with mutations in transcription factor genes (*AhR*, *Fxr*, *Hnf1a*, *Hnf4a*) that regulate xenobiotic metabolism.

- Lee and Riddick found that *Ahr* inactivation leads to dysregulation of hepatic GH signaling components and suppression of STAT5b target genes *Cyp2d9* and *Mup2* [71].
- Compared to wild-type controls, dwarf *Ghrhr* mutant mice (Little mice) exhibit increased expression and DNA binding activity of FXR protein [73]. GH treatment decreases hepatic expression of FXR and the female-specific genes *Abcb1a*, *Fmo3* and *Gsta2* in both wild-type and Little mice. FXR positively controls *Abcb1a*, *Fmo3*, and *Gsta2* expression through direct interactions with the response elements in these genes. The authors speculate that increased FXR signaling may play a critical role in the lifespan extension observed in Little mice [73]. These findings however, do not explain how knockout of *Fxr* leads to feminization.
- HNF1α is a strong activator of the male-specific rat *CYP2C11* gene promoter, where it suppresses inhibitory effects of HNF3β on STAT5 function [74].
- STAT5b and HNF4α can co-regulate sex-specific liver genes by mechanisms that are primarily active in male liver. STAT5b and HNF4a exhibit mutual signaling cross-talk [75]. Liver-specific deletion of the *Hnf4a* gene has a sex-dependent impact on gene expression in the liver [76]. In an analysis of the global DNaseI hypersensitivity (i.e., chromatin accessibility) of the mouse liver genome, a HNF4α-like motif is most highly enriched in sites within 10 kb of sex-specific genes [77].

In summary, this analysis identified genes that affect the GH-STAT5b axis. Other genes identified in this study await further characterization of how they might interact with, and when altered disrupt the normal regulation of the HPL-GH axis.

## Discussion

A biomarker-based approach that assesses the activation status of STAT5b in mouse liver was used to identify diverse factors that disrupt the hypothalamo-pituitary-liver (HPL) GH axis. A STAT5b biomarker gene set comprised of 144 genes was built using liver microarray profiles from male vs. female comparisons and those between STAT5b-null vs. wild-type mice. These genes 1) are dependent on STAT5b for sex-dimorphic expression (Fig 2A), 2) are responsive to testosterone based on published studies annotated in the Comparative Toxicogenomics Database (CTD) (Fig 2B), and 3) include many genes involved in sex-dependent xenobiotic metabolism (Fig 2C).

To screen for factors that lead to alterations of STAT5b function, we compared the STAT5b biomarker set to a gene expression compendium of annotated biosets using the fold-change rank-based nonparametric Running Fisher algorithm [34], which is analogous to the Gene Set Enrichment Analysis (GSEA) method [43, 44]. The gene biomarker reliably predicted STAT5b activation or suppression, with a balanced accuracy of 99% and 97%, respectively (Fig 3C). This high degree of accuracy was in spite of our use of positive or negative controls from different experiments carried out in different labs that queried gene expression using different microarray platforms. Although the high degree of accuracy could be due in part to the fact that the STAT5b gene response is dramatic, the combination of expert-generated gene biomarkers and the Running Fisher algorithm appears to be a generally useful strategy to assess modulation of other transcription factors and pathways, as shown by its proven utility for identification of modulators of the xenobiotic-activated receptors AhR, CAR and PPARα [31–33].

The STAT5b biomarker gene set readily identified biosets expected to show changes in liver STAT5b function. These included biosets describing masculinization of male mouse liver at puberty (~30 days of age) (Fig 5C) and the subtle feminization of the liver in aging males (Fig 5D). Little, if any, changes in STAT5b function were observed in in vitro comparisons due to the requirement for an intact HPL axis that regulates the male-specific pulsatile GH release from the pituitary (Fig 3B). The STAT5b biomarker correctly predicted STAT5b effects caused by modulation at multiple nodes in the HPL-GH axis. Testosterone levels determine the pattern of GH secretion from the pituitary [6, 7] and biosets in the compendium showed that castration disrupts and testosterone injection restores the male-specific pattern of liver STAT5b activation (described in greater detail in Oshida et al. [30]). Consistent with this finding, disruption of the *androgen receptor* gene results in a microarray profile consistent with feminization of liver gene expression [47]. Feminization was also observed in mutant mice in which GH synthesis and secretion (*Ghrhr*, *Pit1*, *Pou1f1*) or liver GH signal transduction (*Ghr*) was disrupted (Fig 6A). Biosets from fasted mice also showed feminization (S1 File), consistent with observed effects of fasting on GH secretion, including a decrease in the mass of GH per burst, a decrease in the pulsatile and total GH secretion rate, and an increase in the irregularity of the GH pulses [78]. Thus, the STAT5b biomarker developed here is a useful tool to determine the integrative effects of diverse factors through disruption of one or multiple nodes on the HPL-GH axis.

We examined the utility of the biomarker in identifying STAT5b modulation in tissues other than the liver. In a preliminary analysis of 249 biosets from 59 studies derived from mouse testis and ovaries, we did not find any biosets in which the STAT5b biomarker gene set was significantly altered. This likely reflects tissue-specific differences in the expression and regulation of STAT5b gene targets. Thus, the biomarker that we developed in this study for liver STAT5b function is most likely useful for identification of factors that affect STAT5b in liver but not other tissues.

**Fig 7:**
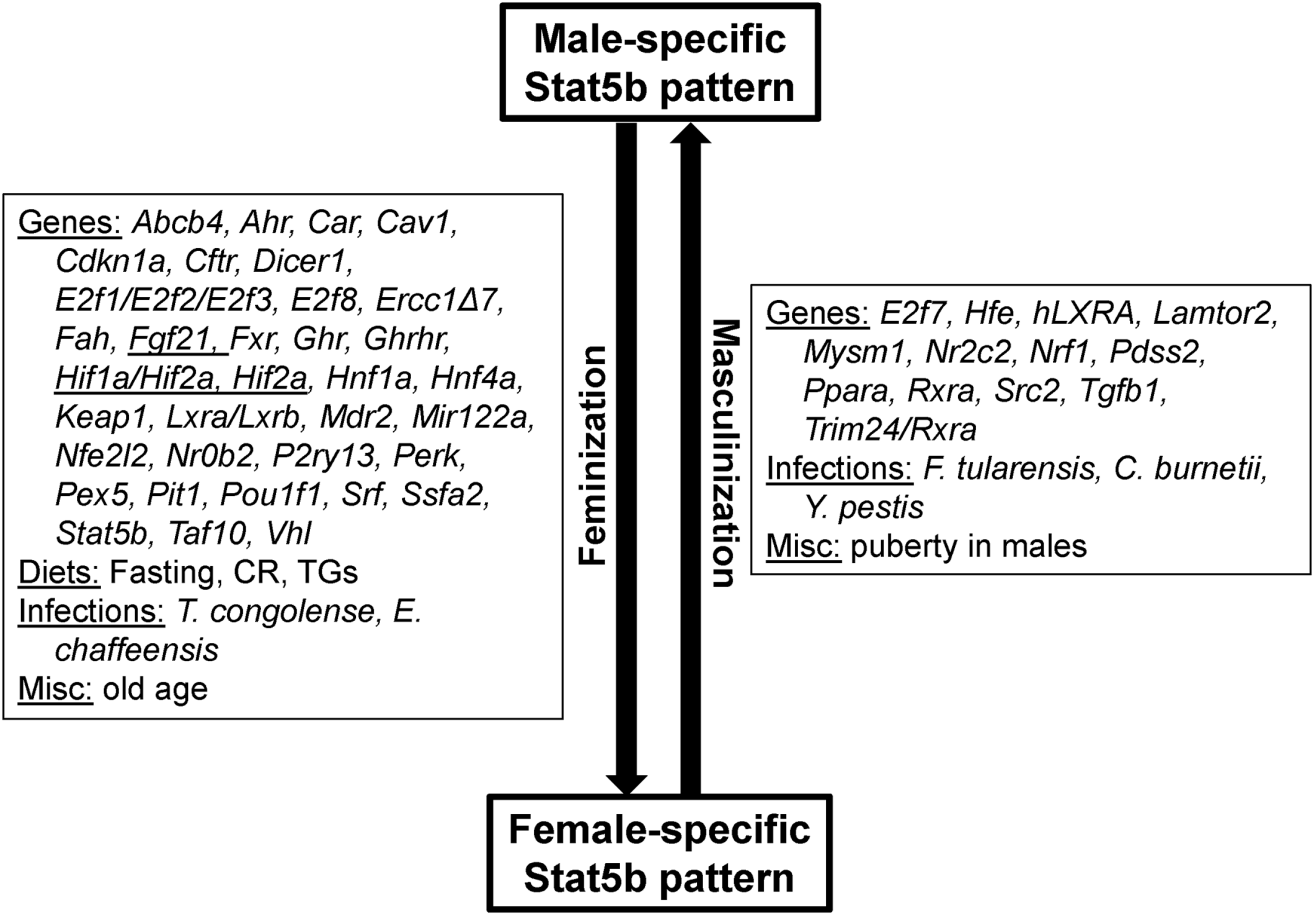
Summary of factors that masculinize or feminize the liver transcriptome identified in this study. For the listed genes, most of the observations of feminization or masculinization occur when the gene was disrupted. Mouse genes that when overexpressed result in the effects shown are underlined. A number of mouse models expressed human genes, which are indicated as capitalized and preceded by “h”. Abbreviations: CR, caloric restriction; TG, triglycerides.

Comparison of the STAT5b biomarker to a compendium of liver gene expression biosets demonstrated that disruption of the HPL-GH axis, as assessed by changes in STAT5b-dependent sex-biased gene expression, is a relatively common occurrence in mouse liver. Livers of male mice appear to be particularly sensitive to feminization. In males, out of the 496 biosets evaluated, ~29% were feminized, whereas only ~5% were masculinized. In females, out of the 174 biosets evaluated, biosets were almost evenly distributed between those that were masculinized (~11%) or feminized (~13%). Factors that disrupted STAT5b regulation and caused feminization included fasting, caloric restriction, synthetic triglycerides, and infections (*Trypanosoma congolense*, *Ehrlichia chaffeensis*), whereas masculinization was caused by three infectious agents (*Francisella tularensis*, *Coxiella burnetii*, *Yersinia pestis*) (Fig 7). Strikingly, for 70 genes, genetic alteration (i.e., null alleles or gene overexpression) was found to alter liver STAT5b function. These genes are involved in GH secretion or signaling, as discussed above, but also include nuclear receptors (*Car*, *Fxr*, *Hnf4a*, *Lxra/Lxrb*, *Nr0b2*, *Nr2c2*, *Ppara*, *Rxra*), other transcription factors (*Ahr*, *E2f1/E2f2/E2f3*, *E2f8*, *Hnf1a*, *Nrf1*, *Src2*, *Srf*, *Taf10*, *Trim24*), Nrf2 signaling components (*Keap1*, *Nfe2l2*), and miscellaneous genes (*Abcb4*, *Cav1*, *Cdkn1a*, *Cftr*, *Dicer1*, *Ercc1Δ7*, *Fah*, *Hfe*, *Lamtor2*, *Mdr2*, *Mir122a*, *Mysm1*, *P2ry13*, *Pdss2*, *Perk*, *Pex5*, *Ssfa2*, *Tgfb1*, *Vhl*). The fact that disruption of STAT5b function appears to be a common occurrence may reflect the multiple ways in which the HPL-GH axis can be disrupted, and/or the sensitivity of some of the nodes to perturbation. How the factors identified in the present study disrupt the HPL-GH axis remains to be determined in future studies.

## Supporting Information

S1 File Supplemental information. Contains 1) determination of the relationship between number of overlapping genes and Running Fisher Algorithm p-value; 2) effects of method used to derive gene lists on STAT5b predictions; 3) effects of diets on STAT5b; 4) effects of infections on STAT5b; 5) relationships between STAT5b and expression of components of bioactive IGF-1.
(DOCX)

S2 File List of genes in the STAT5b biomarker. (XLSX)

## Abbreviations

CTD: comparative toxicogenomics database
GH: growth hormone
HPL: hypothalamic-pituitary-liver
STAT5b: signal transducer and activator of transcription 5b
