# Supplementary material for: Disruption of STAT5b-Regulated Sexual Dimorphism of the Liver Transcriptome by Diverse Factors Is a Common Event
Source: PLoS One. 2016 Mar 9;11(3):e0148308. doi: 10.1371/journal.pone.0148308 (PMC4784905; doi:10.1371/journal.pone.0148308)
Supplement: S1 File — Contains 1) determination of the relationship between number of overlapping genes and Running Fisher Algorithm p-value; 2) effects of method used to derive gene lists on STAT5b predictions; 3) effects of diets on STAT5b; 4) effects of infections on STAT5b; 5) relationships between STAT5b and expression of components of bioactive IGF-1. (DOCX) [file pone.0148308.s001.docx]

**Supplemental File 1 to**

**Disruption of STAT5b-regulated Sexual Dimorphism of the Liver Transcriptome by Diverse Factors is a Common Event**

by

Keiyu Oshida, Naresh Vasani, David J. Waxman and J. Christopher Corton

**Determination of the relationship between number of overlapping genes and Running Fisher’s Algorithm p-value.**

The relationship between number of genes that overlapped with the STAT5b signature and the p-values of the similarity were examined. A plot of the –log(p-value) vs the number of overlapping genes for ~2400 biosets shows a pattern similar in appearance to a typical volcano plot of microarray data (**Supplemental Figure 1**). (It should be noted that a volcano plot X and Y axes are fold-change and –log(p-value), respectively.) In the present case, the deviation from a –log(p-value) of 0 tended to increase with increase in the number of overlapping genes. The average number of overlapping genes per bioset was 30.0. The average number of overlapping genes for biosets with significant masculinization or feminization was 54 (range 4 -128) or 48 (range 6 - 135), respectively, greater than the average number of overlapping genes in biosets that were not significant (19; range, 1 - 101). The lower number of overlapping genes in biosets that were not significant was not surprising given the requirement for some overlap between the signature and the bioset for the Running Fishers algorithm to derive a p-value.


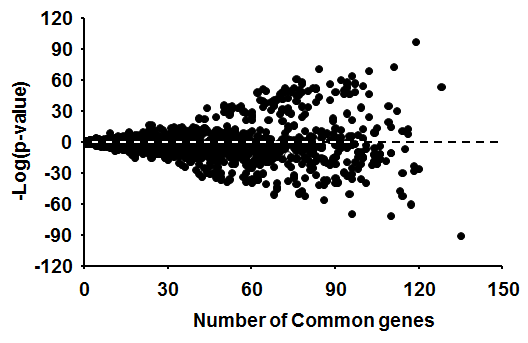


**Supplemental Figure 1. Relationships between number of overlapping genes with STAT5b signature and the Running Fishers Algorithm similarity p-values.**

Plot of –Log(p-value) vs. number of genes that overlap with the STAT5b signature.

**Effects of method used to derive gene lists on Running Fishers Algorithm p-value.**

Considering the gene lists derived in our lab were derived using different computational approaches than those used in the NextBio pipeline (see Methods section of the paper), we compared the predictions of STAT5b activation or suppression using pairs of biosets derived by the two methods. One hundred thirty three biosets from 16 studies were compared directly. A plot of the number of genes that overlapped with the STAT5b signature is shown in **Supplemental Figure 2, left**. In general, there was good concordance in the number of genes derived by the two methods, although the NextBio pipeline allowed for greater numbers of genes, most likely due to the purposeful absence of a multiple test correction (Kuperschmidt et al., 2010). Despite these differences in the number of overlapping genes derived between the two methods, the concordance of the p-values was very good (**Supplemental Figure 2, right**). Out of the 133 biosets there were 10 biosets that were called significant using EPA methods that were not significant when derived using the NextBio methods. Alternatively, there were 8 biosets that were called significant using NextBio methods that were not significant using our methods. Overall the concordance was 86%. Thus, the choice of the method to derive the lists of significant genes does not appear to be a significant factor in identifying biosets that exhibited activation or suppression of STAT5b.


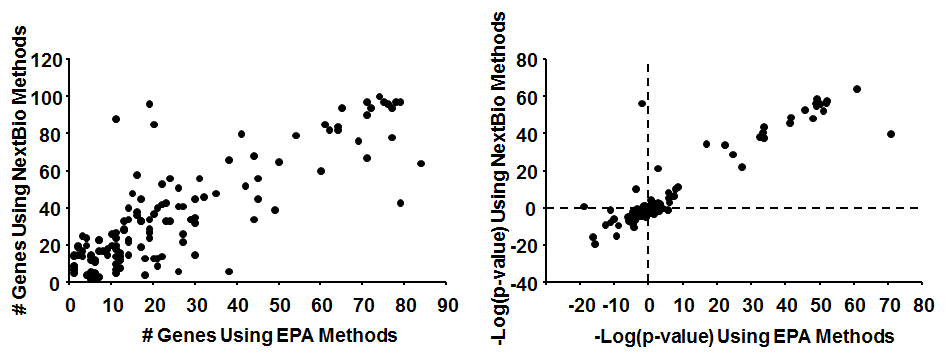


**Supplemental Figure 2.** Relationships between overlap with the STAT5b signature for biosets of statistically filtered genes derived by the EPA or NextBio methods.

A. Number of genes identified as significantly altered.

B. Relationships between predictions of masculinization or feminization.

**Effects of diets on liver STAT5b function.**

The effects of fasting and caloric restriction on liver STAT5b function were examined. Four of the 19 biosets, in which mice were fasted for up to 48 hr, exhibited feminization and all but one of these biosets could be attributed to experiments in male mice (**Supplemental Figure 3A**). Additional biosets showed feminization that in some cases approached significance. None of the biosets showed significant masculinization. The effect of fasting on STAT5b function is consistent with a study that quantitated the effects of fasting on GH secretion and showed a decrease in the mass of GH per burst, decreases in the pulsatile and total GH secretion rate, and an increase in the irregularity of the GH pulses (Steyn et al., 2011). Out of the 6 biosets in which the effects of long term (> 48 hr) caloric restriction were examined, 3 biosets showed significant feminization and none reached statistical significance for masculinization (**Supplemental Figure 3B**). Caloric restriction has been shown to feminize the male liver transcriptome in a graded (0, 15, 30, or 40%) one month caloric restriction (CR) model (Fu and Klaassen, 2014). These results indicate that the STAT5b signature predicts the known feminization caused by fasting and caloric restriction.

Caloric restriction and fasting result in mobilization of triglycerides from fat stores for catabolism in the liver. The effect of triglyceride exposure itself was examined to determine if the exposure feminizes the liver. Six hours after gavage treatment, the liver was significantly feminized by synthetic triglycerides composed of C10:0, C18:3, and C22:6 fatty acids in wild-type mice and triglycerides composed of C10:0 and C18:3 in PPARα-null mice (GSE8396) (**Supplemental Figure 3C**). Overall, these findings support a model in which increases in mobilized triglycerides suppress STAT5b function, possibly through decreases in the secretion of GH. Support for this hypothesis comes from the ability of increased levels of free fatty acids to suppress GH secretion (Maccario et al., 1994; Alvarez et al., 1991).


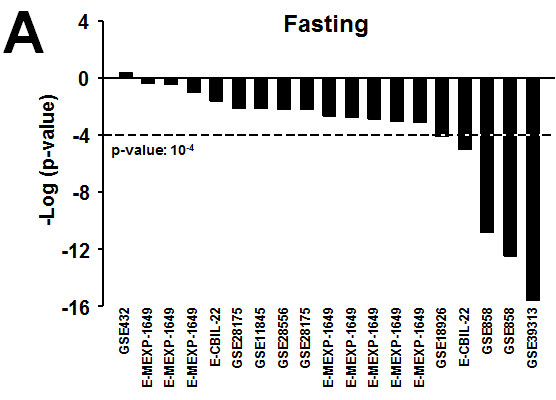

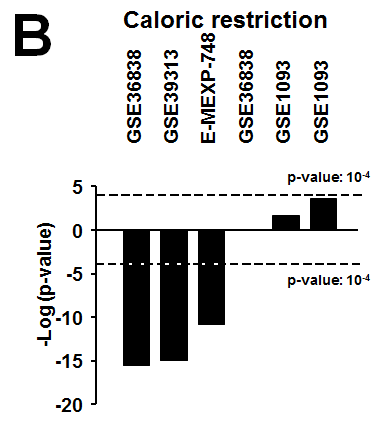

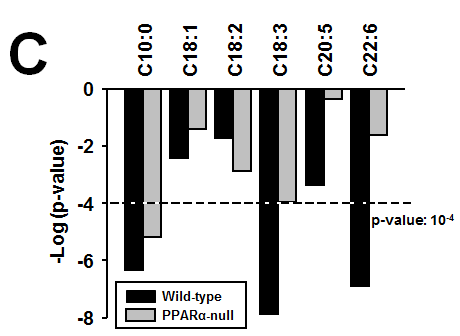


**Supplemental Figure 3. Effects of caloric restriction, fasting, and triglyceride exposure on STAT5b.**

A. Effects of fasting. The –log(p-value) of the similarity to the STAT5b signature for 19 biosets from mice fasted for up to 24 or 48 hr.

B. Effects of caloric restriction. The –log(p-value) of the similarity to the STAT5b signature for 6 biosets from mice calorically restricted for > 48 hr.

C. Comparison of synthetic triglyceride treatments in wild-type and PPARα-null mice on STAT5b function. Effects of synthetic triglycerides composed of 6 different fatty acids are shown. The data are from GSE8396. Dashed horizontal lines represent the p-value cutoff for significance.

**Impact of infections on STAT5b function**

Given the role of STAT5b in regulating immune responses (Rochman et al., 2009), it was hypothesized that STAT5b would be modulated by infectious agents. Out of a total of 91 biosets examining the effects of infection, masculinization or feminization was observed in 6 and 15 biosets, respectively (**Supplemental Figure 4A**). Three infectious agents caused masculinization. Female mice pretreated with saline or with lipopolysaccharide from *Francisella tularensis* (the gram negative bacterium that is the causal agent of tularemia in humans) were challenged with *F. tularensis* itself (GSE16207). The female livers were masculinized at 48 hr but not 24 hr after exposure (**Supplemental Figure 4B, left**). Intact or castrated male mice infected with *Coxiella burnetii* (the gram negative bacterium that is the causative agent of Q fever) exhibited masculinization (GSE21065) (**Supplemental Figure 4B, middle**). Infected ovariectomized but not intact female livers also exhibited masculinization. Sex-related differences in gene expression have been characterized after *C. burnetii* infection in mice (Textoris et al., 2010). Female mice infected with *Yersinia pestis* (the gram negative cocobacillus bacterium that causes the plague) carrying a lipoprotein mutant gene but not wild-type *Y. pestis* caused masculinization of the liver after 48 hr, but not 12 hr post-infection (GSE18293) (**Supplemental Figure 4B, right**).

Two infectious agents caused feminization of the liver. These include *Trypanosoma congolense,* a unicellular parasitic flagellate protozoa and the major pathogen responsible for the disease nagana. Three strains of mice (with no sex designation) were infected with *T. congolense* and the livers were examined at time points up to 17 days for gene expression changes (bioset E-MEXP-1190). A/J and Balb/c mice but not C57Bl/6 mice exhibited significant feminization of the liver in at least two of the four time points (**Supplemental Figure 4C**). The pattern of feminization corresponds with the sensitivity of the strains to *T. congolense* infection; C57BL/6 mice survive signiﬁcantly longer than A/J or Balb/c mice (Iraqi et al., 2000). *Ehrlichia chaffeensis* is an obligate intracellular gram-negative species of *Rickettsiales* bacteria and the causative agent of human monocytic ehrlichiosis. Infection of *Scid* mice by all three strains of *E. chaffeenisis* in one study (GSE8966) caused feminization after 15 days (p-values = 5.5E-7 to 2.7E-25). Taken together, the studies indicate that infection with gram negative bacteria can lead to activation or suppression of liver STAT5b function, whereas infection with at least one nonbacterial species suppresses STAT5b function. Our analysis identified 5 infectious agents that modulate the activity of liver STAT5b.

The fact that infections agents can have effects on liver STAT5b function is consistent with the role that STAT5b has in immunity. STAT5b is activated by a large number of cytokines (Leonard and O'Shea, 1998; Rochman et al., 2009) and plays a critical role in natural killer cell development and function (Imada et al., 1998; Moriggl et al., 1999). Patients carrying mutations in the *STAT5B* gene often exhibit immune dysfunction that can lead to severe, life-threatening infections and chronic pulmonary disease, consistent with the fact that STAT5b is activated by multiple cytokines involved in immunity (Feigerlova et al., 2013). In the immune system, STAT5b-null mice exhibit decreased interleukin 2-induced interleukin 2 receptor α chain expression in splenocytes (Imada et al., 1998). Lipopolysaccharide (LPS) attenuates GH-induced hepatic insulin-like growth factor I expression by inhibiting JAK2/STAT5 signal transduction and STAT5b DNA binding (Chen et al., 2007). There are 9 biosets from mice treated with lipopolysaccharide in our compendium but only one of these (from GSE33901 in which mice were exposed to LPS for 20 hr) caused feminization of the liver (p-value = 8.3E-5). None caused significant masculinization (data not shown). As most of the lipopolysaccharide treatments were for shorter incubation times, longer exposure times may be necessary for feminization effects to be observed. Overall, the identification of models of infection that have effects on STAT5b function could be used in future studies to determine the molecular basis of the effects and whether manipulation of liver STAT5b activity has an impact on immune responses to infection.


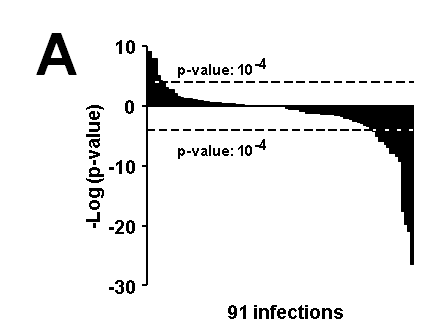

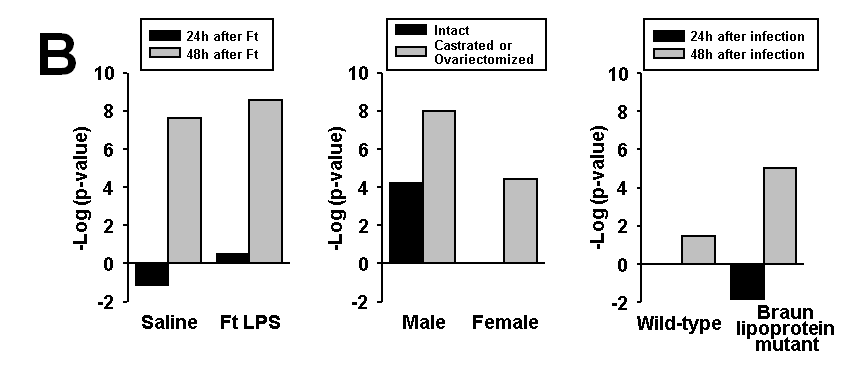


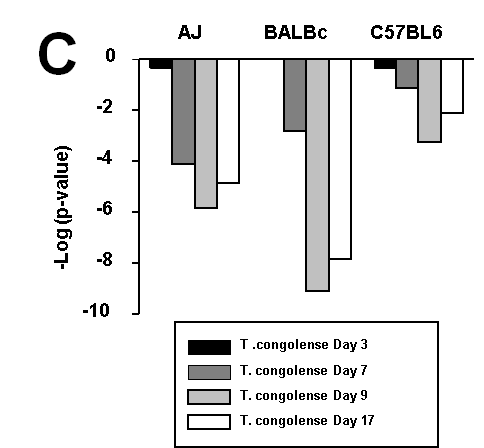


**Supplemental Figure 4. Effects of infectious agents on STAT5b.**

A. Distribution of –log(p-value)s of biosets from mice exposed to infectious agents.

B. Masculinization of the liver transcriptome after infection by *Francisella tularensis* (Ft; left), *Coxiella burnetii* (middle), or *Yersinia pestis* (right). See text for description of the studies.

C. Feminization after infection with *T. congolense* for up to 17 days in three strains of mice.

**Relationships between STAT5b function and expression of components of bioactive IGF-1**

Loss of sex-specific liver gene expression could come about through disruption at multiple points in the HPL GH axis. The regulation of body size by GH is mainly executed by the activation of STAT5b in the liver, which, in turn, regulates the transcription of the Igf1 gene and the acid labile subunit (ALS encoded by Igfals) gene (Ooi et al., 1998; Woelfle et al., 2003a,b). IGF-1 and ALS form a trimeric complex together with IGF-1 binding protein 3 (*Igfbp3*), termed bioactive IGF-1, in the serum (Dai and Baxter, 1994; Jones and Clemmons, 1995), which promotes cellular growth and controls neuroendocrine functions. Bioactive IGF-I negatively regulates GH secretion (Bermann et al., 1994). Deletion of *Igf1* in the liver increases circulating GH levels as a result of the loss of an inhibitory feedback mechanism that regulates pituitary GH release (Sjogren et al., 1999; Wallenius et al., 2001). Similarly, disruption of the *Ghr* gene results in increased GH levels (Barclay et al., 2011). We hypothesized that biosets which had decreased expression of bioactive IGF-I components or *Ghr* in the liver would also exhibit feminization possibly by disruption of male-specific GH secretion.

To address the relationship between the expression of components of bioactive IGF-I and the function of STAT5b, biosets in which *Ghr,* *Igf1, Igfals,* or *Igfbp3* exhibited increased or decreased expression (|fold-change| ≥ 1.5) were examined for changes in STAT5b function. *Ghr, Igf1* and *Igfals* exhibited similar relationships between their expression levels and liver STAT5b activation (**Supplemental Figure 5**). The fact that *Ghr* expression was linked to STAT5b activation status was not surprising, given that *Ghr* is one of the genes in the STAT5b signature. Biosets that had increased expression of *Ghr, Igf1* or *Igfals* tended to exhibit masculinization, whereas biosets that had decreased expression exhibited far greater numbers of feminization compared to masculinization, consistent with our hypothesis. In contrast, increased expression of *Igfbp3* was found in greater numbers of biosets that exhibited feminization than masculinization. Whether the feminization in these biosets is due to higher circulating GH levels, or not, remains to be determined. Overall, these results provide evidence that alterations in the expression of genes that encode components of the bioactive IGF-1 occur in concert with masculinization or feminization, possibly by enforcing or disrupting the male-dependent pattern of GH secretion.


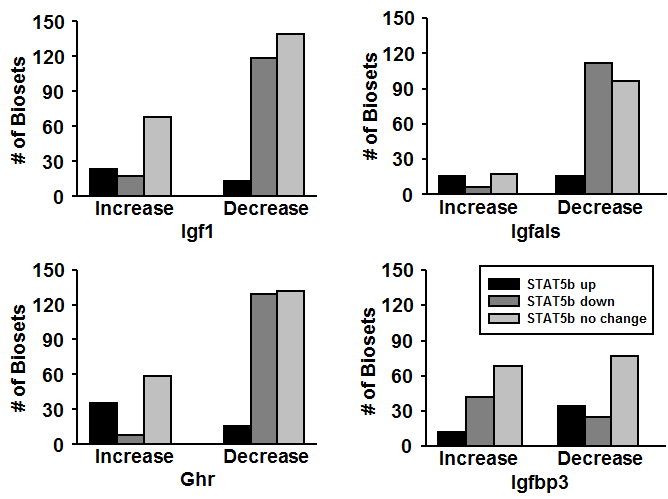


**Supplemental Figure 5. Relationships between STAT5b status and expression of components of bioactive IGF1.**

The expression of the indicated genes involved in GH-mediated induction of bioactive IGF1 was compared to the predictions of effects on STAT5b function. An increase or a decrease in gene expression was defined as ≥ 1.5-fold or ≤ -1.5-fold change in expression, respectively.

**References**

Alvarez CV1, Mallo F, Burguera B, Cacicedo L, Dieguez C, Casanueva FF. Evidence for a direct pituitary inhibition by free fatty acids of in vivo growth hormone responses to growth hormone-releasing hormone in the rat. Neuroendocrinology. 1991 Feb;53(2):185-9.

Barclay JL, Nelson CN, Ishikawa M, Murray LA, Kerr LM, McPhee TR, Powell EE, Waters MJ. GH-dependent STAT5 signaling plays an important role in hepatic lipid metabolism. Endocrinology. 2011 Jan;152(1):181-92.

Bermann M1, Jaffe CA, Tsai W, DeMott-Friberg R, Barkan AL. Negative feedback regulation of pulsatile growth hormone secretion by insulin-like growth factor I. Involvement of hypothalamic somatostatin. J Clin Invest. 1994 Jul;94(1):138-45.

Chen Y, Sun D, Krishnamurthy VM, Rabkin R. Endotoxin attenuates growth hormone-induced hepatic insulin-like growth factor I expression by inhibiting JAK2/STAT5 signal transduction and STAT5b DNA binding. Am J Physiol Endocrinol Metab. 2007 Jun;292(6):E1856-62.

Dai J, Baxter RC. Regulation in vivo of the acid-labile subunit of the rat serum insulin-like growth factor-binding protein complex. Endocrinology. 1994 Dec;135(6):2335-41.

Feigerlova E, Hwa V, Derr MA, Rosenfeld RG. Current issues on molecular diagnosis of GH signaling defects. Endocr Dev. 2013;24:118-27.

Fu ZD, Klaassen CD. Short-term calorie restriction feminizes the mRNA profiles of drug metabolizing enzymes and transporters in livers of mice. Toxicol Appl Pharmacol. 2014 Jan 1;274(1):137-46.

Imada K, Bloom ET, Nakajima H, Horvath-Arcidiacono JA, Udy GB, Davey HW, Leonard WJ. STAT5b is essential for natural killer cell-mediated proliferation and cytolytic activity. J Exp Med. 1998;188:2067–2074.

Iraqi F, Clapcott SJ, Kumari P, Haley CS, Kemp SJ, Teale AJ. Fine mapping of trypanosomiasis resistance loci in murine advanced intercross lines. Mamm Genome. 2000 Aug;11(8):645-8.

Jones JI, Clemmons DR. Insulin-like growth factors and their binding proteins: biological actions. Endocr Rev. 1995 Feb;16(1):3-34.

Leonard WJ, O'Shea JJ. Jaks and STATs: biological implications. Annu Rev Immunol. 1998;16:293–322.

Maccario M1, Procopio M, Loche S, Cappa M, Martina V, Camanni F, Ghigo E. Interaction of free fatty acids and arginine on growth hormone secretion in man. Metabolism. 1994 Feb;43(2):223-6.

Moriggl R, Topham DJ, Teglund S, Sexl V, McKay C, Wang D, Hoffmeyer A, van Deursen J, Sangster MY, Bunting KD, et al. Stat5 is required for IL-2-induced cell cycle progression of peripheral T cells. Immunity. 1999;10:249–259.

Ooi GT1, Hurst KR, Poy MN, Rechler MM, Boisclair YR. Mol Endocrinol. Binding of STAT5a and STAT5b to a single element resembling a gamma-interferon-activated sequence mediates the growth hormone induction of the mouse acid-labile subunit promoter in liver cells. 1998 May;12(5):675-87.

Rochman Y, Spolski R, Leonard WJ. New insights into the regulation of T cells by gamma(c) family cytokines. Nat Rev Immuno. 2009;19:480–490.

Sjögren K, Liu JL, Blad K, Skrtic S, Vidal O, Wallenius V, LeRoith D, Törnell J, Isaksson OG, Jansson JO, Ohlsson C. Liver-derived insulin-like growth factor I (IGF-I) is the principal source of IGF-I in blood but is not required for postnatal body growth in mice. Proc Natl Acad Sci U S A. 1999 Jun 8;96(12):7088-92.

Steyn FJ, Huang L, Ngo ST, Leong JW, Tan HY, Xie TY, Parlow AF, Veldhuis JD, Waters MJ, Chen C. Development of a method for the determination of pulsatile growth hormone secretion in mice. Endocrinology. 2011 Aug;152(8):3165-71.

Textoris J, Ban LH, Capo C, Raoult D, Leone M, Mege JL. Sex-related differences in gene expression following Coxiella burnetii infection in mice: potential role of circadian rhythm. PLoS One. 2010 Aug 13;5(8):e12190. doi: 10.1371/journal.pone.0012190.

Wallenius K, Sjögren K, Peng XD, Park S, Wallenius V, Liu JL, Umaerus M, Wennbo H, Isaksson O, Frohman L, Kineman R, Ohlsson C, Jansson JO. Liver-derived IGF-I regulates GH secretion at the pituitary level in mice. Endocrinology. 2001 Nov;142(11):4762-70.

Waxman DJ, Holloway MG. Sex differences in the expression of hepatic drug metabolizing enzymes. Mol Pharmacol. 2009 Aug;76(2):215-28. doi: 10.1124/mol.109.056705. Epub 2009 May 29.

Woelfle J, Billiard J, Rotwein P. Acute control of insulin-like growth factor-I gene transcription by growth hormone through STAT5b. J Biol Chem. 2003a Jun 20;278(25):22696-702.

Woelfle J, Chia DJ, Rotwein P. Mechanisms of growth hormone (GH) action. Identification of conserved Stat5 binding sites that mediate GH-induced insulin-like growth factor-I gene activation. J Biol Chem. 2003b Dec 19;278(51):51261-6.
